# *CG4928* Is Vital for Renal Function in Fruit Flies and Membrane Potential in Cells: A First In-Depth Characterization of the Putative Solute Carrier UNC93A

**DOI:** 10.3389/fcell.2020.580291

**Published:** 2020-10-14

**Authors:** Mikaela M. Ceder, Tanya Aggarwal, Kimia Hosseini, Varun Maturi, Sourabh Patil, Emelie Perland, Michael J. Williams, Robert Fredriksson

**Affiliations:** ^1^Department of Pharmaceutical Biosciences, Molecular Neuropharmacology, Uppsala University, Uppsala, Sweden; ^2^Department of Pharmacy, Drug Delivery, Uppsala University, Uppsala, Sweden; ^3^Department of Neuroscience, Functional Pharmacology, Uppsala University, Uppsala, Sweden

**Keywords:** solute carrier, putative solute carrier, major facilitator superfamily, *D. melanogaster*, CG4928, UNC93A

## Abstract

The number of transporter proteins that are not fully characterized is immense. Here, we used *Drosophila melanogaster* and human cell lines to perform a first in-depth characterization of CG4928, an ortholog to the human UNC93A, of which little is known. Solute carriers regulate and maintain biochemical pathways important for the body, and malfunctioning transport is associated with multiple diseases. Based on phylogenetic analysis, CG4928 is closely related to human UNC93A and has a secondary and a tertiary protein structure and folding similar to major facilitator superfamily transporters. Ubiquitous knockdown of *CG4928* causes flies to have a reduced secretion rate from the Malpighian tubules; altering potassium content in the body and in the Malpighian tubules, homologous to the renal system; and results in the development of edema. The edema could be rescued by using amiloride, a common diuretic, and by maintaining the flies on ion-free diets. CG4928-overexpressing cells did not facilitate the transport of sugars and amino acids; however, proximity ligation assay revealed that CG4928 co-localized with TASK^1^ channels. Overexpression of CG4928 resulted in induced apoptosis and cytotoxicity, which could be restored when cells were kept in high-sodium media. Furthermore, the basal membrane potential was observed to be disrupted. Taken together, the results indicate that CG4928 is of importance for generating the cellular membrane potential by an unknown manner. However, we speculate that it most likely acts as a regulator or transporter of potassium flows over the membrane.

## Introduction

Transporters, especially solute carriers (SLCs), have been underrepresented in research (Cesar-Razquin et al., 2015). In fact, many transporters remain orphans, and details about their expression and function are still unknown (Perland and Fredriksson, 2017). Today there are around 2,000 genes in humans that encode for proteins that mediate, sense, and/or regulate the transport of different solutes across membranes (Urban et al., 2006). Transporters are vital for biochemical pathways to maintain the cellular concentrations of solutes, to excrete waste products, and to generate electrochemical gradients (Saier, 2000). Malfunctioning transport systems are associated with a disturbance of cellular processes, which cause several diseases and disorders (Zhang et al., 2018), e.g., metabolic disorders (Dupuis et al., 2010) and kidney dysfunction (Kottgen et al., 2010). One of these orphan transporters is UNC93A, which was revealed by protein sequence analysis (Hellsten et al., 2017; Perland and Fredriksson, 2017; Perland et al., 2017a) and genome annotation (Fredriksson et al., 2008) to be a putative SLC of major facilitator superfamily (MFS) type. Members of the MFS are facilitators and secondary active transporters with various expressions, functions, and substrate profiles (Pao et al., 1998; Reddy et al., 2012). Like many SLCs of MFS type, UNC93A is evolutionary conserved (Ceder et al., 2017).

Studies on UNC93A have been performed using *Caenorhabditis elegans* (Levin and Horvitz, 1992; de la Cruz et al., 2003), *Aedes aegypti* (Campbell et al., 2011), *Drosophila melanogaster* (Wang et al., 2004; Stofanko et al., 2008; Mohr et al., 2018; Ceder et al., 2020), *Anguilla anguilla* (Kalujnaia et al., 2007), *Mus musculus* (Ceder et al., 2017, 2020; Perland and Fredriksson, 2017; Perland et al., 2017a), and humans (Liu et al., 2002; Son et al., 2015; Schlosser et al., 2020), but a majority of these studies are not focusing on the localization and/or the function of UNC93A. The ortholog found in *C. elegan*s is suggested to be a regulatory protein of a potassium channel (de la Cruz et al., 2003). Interestingly, there is also evidence that UNC93A is a membrane-bound protein (Levin and Horvitz, 1992; Liu et al., 2002; Ceder et al., 2017) with a 2D topology resembling an authentic transporter according to predictions (Ceder et al., 2017; Perland et al., 2017a). In *D. melanogaster*, the ortholog *CG4928* was found to be highly expressed in the Malpighian tubules, equivalent to the human kidneys (Wang et al., 2004), to be important for hemocyte development (Stofanko et al., 2008), and to be one of several genes that confer manganese toxicity resistance (Mohr et al., 2018). Furthermore, the human UNC93A was recently identified as a metabolite-associated locus in chronic kidney disease patients (Schlosser et al., 2020).

Recently, we confirmed that UNC93A is evolutionary conserved in mammals and found that it is expressed in metabolic active organs such as the brain and the kidneys. Furthermore, *Unc93a* and *CG4928* are affected by dietary restrictions and starvation in mice and fruit flies (Ceder et al., 2017, 2020). The role of UNC93A and its implications in processes, such as homeostasis and pathology, are not completely known, and to explore its role in humans would be difficult and time consuming. To address these issues and to perform in-depth characterization, we first used the well-studied and simpler organism *D. melanogaster* and cell lines. In this paper, we aimed to characterize the ortholog protein of UNC93A. RNA interference (RNAi) was used to knock down *CG4928* expression and to monitor phenotypic data. We found that *CG4928* is crucial for renal function in the fly. Second, we performed proximity ligation assay (PLA) and confirmed that UNC93A co-localized with TASK^1^ channels. Next, CG4928 was overexpressed in human HEK293T cells to study the substrate profile. We found that CG4928 does not facilitate the transport of sugars and amino acids but induces apoptosis and cytotoxicity as well as disrupts the basal membrane potential. Taken together, the results indicate that CG4928, and possibly also UNC93A, has an impact on ion homeostasis *via* interactions with TASK^1^ channels and/or by facilitating the movement of ions over the membrane.

## Materials and Methods

### Fly Work

#### Fly Stocks and Maintenance

*CG4928* expression was visualized by crossing *CG4928-Gal4* (stock no: 104449, Kyoto Stock Center) and *Pin/Pin-UAS-GFP* (derived from *yw;Pin/CyO*, *UAS-mCG8-GFP* stock no: 5130, Bloomington). For functional characterization, two *CG4928 RNAi* lines (*CG4928-RNAi-UAS* stock nos: 103750 and 6143, VDRC) were used and crossed to the ubiquitous *da-Gal4* (kindly donated by Prof. D. Nässel, Stockholm University), *c601-Gal4* (stock no: 30844, Bloomington), *Uro-Gal4*, *c42-Gal4*, *c324-Gal4*, and *c724-Gal4* (all kindly donated by Prof. J.A. Dow, Glasgow University). *CG4928 RNAi* lines have one reported off-target gene encoding tryptophan hydroxylase (*CG9122*) that was accounted for. W^1118^ (stock no: 5905, Bloomington) was used for control crosses.

Stocks and crosses were maintained at 25°C with 60% humidity on a 12:12-h light/dark cycle; the exceptions for crosses from *CG4928 RNAi GD* and *da-Gal4* were kept at 18°C. Offspring were raised at 28°C for 5 days before the experiments. All flies, unless otherwise stated, had free access to enriched Jazz mix standard fly food (Fisher Scientific).

#### Gene Expression Analysis With GFP

Adult flies were dissected to study the gene expression pattern in Malpighian tubules. To induce *CG4928* cells to express green fluorescent protein (GFP), the *Pin/Pin-UAS-GFP* and the enhancer trap *CG4928-GAL4* lines were crossed. Flies were fixed in 4% formaldehyde for 1 h, followed by washing five times in 1 × phosphate-buffered saline with Tween-20 (PBST; 137 mM NaCl, 2.7 mM KCl, 10 mM Na_2_HPO_4_, 1.8 mM KH_2_PO_4_, and 0.1% Tween-20, Sigma Aldrich). Dissection was performed in 1 × PBST on silicon-coated petri dishes. Tissues were mounted on Superfrost Plus slides in 50% glycerol solution. Images were acquired with an Olympus microscope BX55 with an Olympus DP73 camera and the cellSens Dimension v1.14 (Olympus). Images were then handled using ImageJ, Fiji edition (Schindelin et al., 2012).

#### Fly and Malpighian Tubules Imaging

Flies were quickly frozen in −80°C and imaged using a Leica M125 microscope with a ProgRes C14 plus camera (Jenoptik) and the ProgRes CapturePro 2.8 Jenoptik Optical system. The Malpighian tubules from female and male adult flies were dissected and mounted in 50% glycerol before imaging. Images were handled using ImageJ, Fiji edition (Schindelin et al., 2012).

#### Secretion Assay (Ramsay Assay)

Female flies (*n* = 50) were collected, anesthetized, and dissected. The procedure was performed as described in Schellinger and Rodan (2015). Briefly, the mounted tubules were set up to leak for 2 h before the droplets were measured using an ocular micrometer where one pitch = 0.07 mm. The secretion rate was calculated according to *V* = Π*d*^3^/6. The average secretion rate (±SD) was plotted, and differences were calculated using one-way ANOVA with Bonferroni’s corrections (^∗^*p* < 0.05, ^∗∗^*p* < 0.01, ^∗∗∗^*p* < 0.001) using GraphPad Prism, version 5.

#### Body and Hemolymph Measurements

Adult flies (*n* = 30 per replicate) were collected in pre-weighted 1.5-ml Eppendorf tubes, and body weight was measured using a precision balance (VWR). Flies were then transferred to 0.5-ml Eppendorf tubes, with a hole at the bottom of each tube and placed in 1.5-ml Eppendorf tubes. Hemolymph was collected by centrifugation for 25 min at room temperature at 6,000 × *g* before the hemolymph weight was measured using a precision balance (VWR). The procedure was repeated six to 10 times for each genotype, and the weight was corrected against the number of flies. Graphs represent mean (±SD), and differences were calculated with Kruskal–Wallis and Dunn’s correction (^∗^*p* < 0.05, ^∗∗^*p* > 0.01, ^∗∗∗^*p* > 0.001) using GraphPad Prism, version 5.

#### Sodium, Potassium, and Osmolality Measurements

Hemolymph samples, 20 μl in total (*da-Gal4 > CG4928 RNAi*, *n* = 500; *da-Gal4 > w^1118^*, *n* = 800, *w^1118^ > CG4928 RNAi*, *n* = 800), were collected to measure osmolality and sodium and potassium concentrations. Osmolality was measured using an osmometer (Fiske 210 micro-sample Osmometer Norwood). Concentrations were determined using flame spectrophotometry (model IL543, Instrumentation Lab). The percentages for each genotype were calculated and presented in graphs generated using GraphPad Prism, version 5.

#### Element Analysis

Malpighian tubules from 3-day-old females (*da-Gal4 > CG4928 RNAi*, *da-Gal4 > w^1118^*, and *w^1118^ > CG4928 RNAi*) were dissected, pooled, and frozen in −80°C until analysis. A total of three replicates per genotype were collected, each replicate containing 60 Malpighian tubules. Analysis was performed using inductive coupled plasma sector field mass spectrometry (ICP-SFMS) to analyze a total of 69 elements (ions) in each sample. A control sample was used to measure background in the collecting media. The data were within group, normalized using geometric mean before means (±SD) were calculated and outliers were removed. GraphPad Prism, version 5, was used to perform one-way ANOVA using unpaired *t*-test with Bonferroni’s multiple correction (adjusted *p*-values: ^∗^*p* < 0.0492, ^∗∗^*p* < 0.0099, ^∗∗∗^*p* < 0.0001). The observed data points for boron, cadmium, cobalt, and potassium, which were the only elements found to be altered, are plotted using scatter plot, where the line displays the mean value. The remaining elements are summarized in Supplementary Table S1 with abbreviation, full name of the element, and mean (±SD).

#### RNA Extraction and cDNA Synthesis

Total RNA and cDNA were acquired as described in Chomczynski and Sacchi (1987) and Williams et al. (2016b).

##### RNA extraction

Briefly, flies (*n* = 10 flies per replicate) were homogenized in 60 μl Trizol (Invitrogen). Additional 650 μl Trizol was added followed by incubation for 5 min at room temperature. Then, 160 μl of chloroform (Sigma Aldrich) was added, and the samples were shaken and incubated for 4 min, followed by centrifugation at 14,000 rpm (microcentrifuge from Thermo Fisher Scientific, 24 × 1.5/2.0 ml rotor) at 4°C for 12 min. The upper phase was transferred to a new Eppendorf tube and precipitated with 400 μl isopropanol (Sigma Aldrich) before storing at −20°C for 30 min before centrifugation. The supernatant was discarded, and the RNA pellet was washed three times in 75% ethanol (Sigma Aldrich) and spun for 5 min. During the second wash, DNase treatment (Thermo Scientific, DNase I, RNase Free, 1 U/μl) was added. The pellet was air-dried for 15 min before dissolving in 20 μl of RNAse-free water. The concentration was measured using a ND-1000 spectrophotometer (NanoDrop Technologies).

##### cDNA synthesis

Two micrograms of RNA template was used, and cDNA was synthesized with High Capacity RNA-to-cDNA kit (Applied Biosystems) according to the manufacturer’s instructions. The samples were diluted to approximately 10 ng/μl.

#### Primer Design and Quantitative Real-Time PCR

The primers were designed, and qPCR was performed as described in Ceder et al. (2017). The following housekeeping genes were used: *rp49* forward 5′-*cacaccaaatcttacaaaatgtgtga*-3′ and reverse 5′-*aatccggccttgcacatg*-3′, *rpl11* forward 5′-*ccatcggtatctatggtctgga*-3′ and reverse 5′-*catcgtatttctgctggaacc*-3′, and *Actin42a* forward 5′-*caacacttccgctccttc*-3′ and reverse 5′-*gaacacaatatggtttgctt*-3′, *CG4928* forward 5′-*cggattcgttattgccta*-3′ and reverse 5′-*agtacagccagcagaatg*-3′, *CG9122* forward 5′-*gtggctctacaggagtgg*-3′ and reverse 5′-*cgcatgtggtggaatccttttta*-3′, *Pxt* forward 5′-*atgcgagggtggtgtagtgg*-3′ and reverse 5′-*tcggagttggtcacacaggag*-3′, *Rpt4R* forward 5′-*ctgaaatcggtggacttggt*-3′ and reverse 5′-*aagaaattggcgtccatctg*-3′, and *OATP58Db* forward 5′-*ctcactgtgccatgaaaa*-3′ and reverse 5′-*gcgtagaagagggaacac*-3′. Ct values were obtained using the MyIQ5 software, and primer efficiency was calculated using LinRegPCR software. Normalization was performed against three housekeeping genes (*Actin42a*, *Rpl11*, and *Rp49*). The relative mRNA expression (±SEM) was plotted, gene expression was compared with both controls, and Driver control was set to 100%. Expression differences were calculated using one-way ANOVA and Bonferroni’s correction (^∗^*p* < 0.05, ^∗∗^*p* < 0.01, ^∗∗∗^*p* < 0.001) using GraphPad Prism, version 5.

#### Activity and Starvation Resistance Assay

Activity and starvation assay was performed as described in Artero et al. (1998) and Williams et al. (2016a). In short, adult male flies (*n* = 30) were transferred to 5-mm glass tubes, prepared with 1% agarose, and contained in *Drosophila* activity monitoring systems (DAMS) until the last laser-beam break. Trikinetics software was used to collect bin data; last laser-beam crossing per fly was defined as the timepoint of death. GraphPad Prism, version 5, was used to generate a box plot that represents median (±q1 and q3), and one-way ANOVA with Bonferroni’s correction was performed as statistical analysis (^∗^*p* < 0.05, ^∗∗^*p* < 0.01, ^∗∗∗^*p* < 0.001).

#### Capillary Feeding Assay

The method was conducted as described in Hua et al. (2010) and Williams et al. (2014). Briefly, male adult flies (*n* = 5, per replicate) were transferred to vials supplied with 1% agarose (Conda) and sealed with parafilm. A capillary glass tube (5 μl, VWR International) filled with liquid food (5% sucrose, 5% yeast extract, and 0.5% food-coloring dye) was added to the vial. Initial and final food levels were marked to define the daily food intake. The experiments were carried out at room temperature for 24 h. Food intake per genotype (*n* = 8–10, with five flies in each) was monitored, and the average food intake in nanoliters (±SEM) is presented in the graph. Differences were calculated using one-way ANOVA with Bonferroni’s corrections (^∗^*p* < 0.05, ^∗∗^*p* < 0.01, ^∗∗∗^*p* < 0.001) using GraphPad Prism, version 5.

#### Nutrient Quantification

##### Triacylglyceride (TAG) assay

Male flies were kept on 0, 12, and 24 h of starvation before they were euthanized. Six replicates were made, with five flies in each. The samples were homogenized in 1 × PBST on ice, and 10 μl was collected and stored at −80°C to be used later for protein content measurement. The samples were heat-inactivated at 70°C for 10 min before incubation at 37°C for 45 min with either triglyceride reagent (Sigma Aldrich) or 1 × PBST. The samples were transferred to a 96-well plate, and free glycerol reagent (Sigma Aldrich) was added for 5 min at 37°C. Absorbance was recorded using a multiscan microplate spectrophotometer (Multiskan GO; Thermo Scientific) at 540 nm. TAG was determined by subtracting the amount of free glycerol in the PBST-treated sample from the total glycerol present in the sample treated with triglyceride reagent. Glycerol standard solution (Sigma Aldrich) was used to make equivalent standards.

##### Protein quantification

Total protein was quantified with a protein quantification kit—rapid (Sigma Aldrich) according to the manufacturer’s protocol. Briefly, the homogenates were plated in a 96-well plate in replicates of three, as well as the bovine serum albumin (BSA) standards, and incubated with Coomassie brilliant blue G. Absorbance was measured at 595 nm. Protein concentration was obtained by plotting the calibration curve between the BSA concentration and the value of absorbance.

##### Sugar assay

Male flies were starved for 0, 12, and 24 h before they were euthanized. Ten replicates per genotype, with 10 flies in each, were collected. Hemolymph was collected after decapitation. The flies were weighed, placed in 1 × phosphate-buffered saline (PBS; pH 7.4) and centrifuged. Hemolymph was used to measure the circulating sugars: glucose and trehalose (Sigma Aldrich). To measure the stored sugars (glycogen and trehalose), the bodies were homogenized in 1 × PBS and centrifuged at 12,000 × *g* at 4°C for 15 min, resulting in a body supernatant. For final analysis, the extracted substrates were converted to glucose. Trehalose was converted by porcine kidney trehalase (Sigma Aldrich) incubation overnight at 37°C and glycogen by amyloglycosidase from *Aspergillus niger* (Sigma Aldrich) overnight at 25°C. The glucose levels were quantified using the glucose assay kit with glucose oxidase and peroxidase (Liquick Cor-Glucose diagnostic kit; Cormay), according to the manufacturer’s protocol. Absorbance was measured at 500 nm on a multiscan microplate spectrophotometer (Multiskan GO; Thermo Scientific) and converted to a millimolar concentration of glucose using linear regression obtained by a calibration curve of known glucose concentrations. The glucose measurements are presented in their original unit after conversion.

GraphPad Prism, version 5, was used to generate graphs that represent mean (±SD) and to calculate differences using one-way ANOVA with Bonferroni’s corrections (^∗^*p* < 0.05, ^∗∗^*p* > 0.01, ^∗∗∗^*p* > 0.001).

#### Diuretics, Sugar Diets, and High-Ion Diets—Attempts to Rescue the Phenotype

Male flies (*n* = 30 per replicate, 6 to 10 replicates per genotype) were kept on diuretics, different sugar diets, and high-ion diets for 5 days before they were collected and euthanized. The following diuretics and control diets were used: furosemide (25 mg/ml, Sigma Aldrich) dissolved in acetone (Sigma Aldrich), amiloride (25 mg/ml, Sigma Aldrich) dissolved in water, mannitol (25 mg/ml, Sigma Aldrich) dissolved in water, and acetone-supplemented food. The sugar diets were made with either 40 and 10 g sucrose or glucose (Sigma Aldrich). All sugar diets contained 10 g yeast (protein, Merck Millipore) and were made on deionized water. Diets enriched in NaCl (0.1 mg/ml, Sigma Aldrich) and KCl (0.1 mg/ml, Sigma Aldrich) were made by adding the ion solution to the standard Jazz mix food. Flies raised on untreated standard Jazz mix food were used as control. Body and hemolymph weights were measured as described previously, and photos were taken with a Leica M125 microscope with a ProgRes C14 plus camera (Jenoptik) and the ProgRes CapturePro 2.8 Jenoptik Optical system. The images were handled using ImageJ, Fiji edition (Schindelin et al., 2012). Graphs represent mean (±SD), and differences were calculated with Kruskal–Wallis and Dunn’s correction (^∗^*p* < 0.05, ^∗∗^*p* > 0.01, ^∗∗∗^*p* > 0.001) using GraphPad Prism, version 5.

#### RNA Sequencing

mRNA was separated from total RNA using Dynabeads mRNA Purification kit (Thermo Scientific) according to the manufacturer’s protocol. Poly-A selected RNA was treated with RNaseIII according to the Ion Total RNA-Seq protocol (Thermo Fisher) and purified with Magnetic Bead Cleanup Module (Thermo Fisher). The size and the quantity of the RNA fragments were assessed on Agilent 2100 Bioanalyzer system (RNA 6000 Pico kit, Agilent Technologies) before proceeding to library preparation, using the Ion Total RNA-Seq kit for the AB Library Builder System (Thermo Fisher). The libraries were amplified according to the protocol and purified with Magnetic Bead Cleanup Module (Life Technologies). The samples were then quantified using the Agilent 2100 Bioanalyzer system (High Sensitivity DNA Kit, Agilent Technologies) and pooled, followed by emulsion PCR on the Ion OneTouch 2 system using the Ion PI Hi-Q OT2 200 chemistry (Thermo Fisher). Enriching was conducted using the Ion OneTouch ES (Thermo Fisher). The samples were loaded on an Ion PI v3 Chip (3 samples per chip) and sequenced on the Ion Proton System using Ion PI Hi-Q Sequencing chemistry (200-bp read length, Thermo Fisher).

The reads can be found at the SRA with following PRJNA663443, were mapped against the *D. melanogaster* genome assembly 6.09 using STAR mapper (Dobin et al., 2013). Mapping was done against a genome index generated with the FlyBase GTF annotating file for the 6.09 genome assembly to direct mapping toward annotated genes. A total of 232,620,764 transcripts (Supplementary Dataset S1) were mapped uniquely and used in subsequent analysis. The assembled transcripts were used in CuffLinks and Cuffmerge to obtain a final transcriptome assembly. Subsequently, Cuffquant and Cuffdiff were used to calculate the differential expression, and finally the packaged CummRBound was used in R^1^ to plot the results. The version for all CuffLinks (Trapnell et al., 2010, 2013; Roberts et al., 2011) tools was 2.2.1. Genes were considered to be differentially expressed if they were significantly different (log2 fold change > 1.5 or <−1.5) in *CG4928* knockdown flies compared with both controls in the CuffDiff analysis; the genes are summarized in Supplementary Table S2. DAVID bioinformatics resources were used to investigate clustering and pathway mapping (Huang et al., 2007).

### Proteomics

#### Identification of Proteins Related to UNC93A and Sup-9

A hidden Markov model (Eddy, 2011) for human UNC93A (data version: GRCh38.pep.all) was built and used as described in Ceder et al. (2017) to search the protein data sets listed in Supplementary Table S3. Briefly, the data sets were obtained from Ensembl, version 86 (Cunningham et al., 2015): sequences originating from the same locus and pseudogenes were removed. The longest transcripts were combined in a multiple PSI/TM tcoffee sequence alignment (Notredame et al., 2000), and relationships were concluded with Bayesian approach (Huelsenbeck et al., 2001). The analysis was run *via* the Beagle library (Ayres et al., 2012), five heated and one cold chains, with two runs in parallel (*n* runs = 2). To analyze protein identification, pairwise global sequence alignments were performed using the Needle approach (Li et al., 2015).

The phylogenetic relationship between *C. elegans* Sup-9 and its ortholog protein sequences in *Homo sapiens* and *D. melanogaster* were investigated (Supplementary Dataset S2). Protein sequences were downloaded from Uniprot (UniProt Consortium, 2019). The sequences were aligned using MAFFT (Katoh et al., 2002), and mrBayes 3.2.2 (Huelsenbeck et al., 2001) software was used to generate the tree. The procedure was run on a non-heated chain with two runs in parallel (*n* runs = 2) under the mixed amino acid model with eight gamma categories and invgamma as gamma rates, for a total of 2,000,000 generations.

#### Secondary and Tertiary Structure

Predictions of the *CG4928* structure were modeled using the FBpp0074118 sequence obtained from flybase.org. The secondary structure was modeled using Protter (Omasits et al., 2014), and the tertiary structure was predicted as described in Kelley et al. (2015) and Ceder et al. (2017). The *CG4928* tertiary structure was modeled against glycerol-3-phosphate transporter from *E. coli* (PDB id d1pw4a), with 99.9% confidence and 89% amino acid sequence coverage.

### Cell Work

#### Cell Cultures and Transfection

HEK293T cells, cultured in Dulbecco’s modified Eagle’s medium (DMEM; Thermo Scientific) with 10% fetal bovine serum (FBS) and 1% penicillin and streptomycin, and SH-SY5Y cells, cultured in DMEM: F12 (Thermo Scientific) with 15% FBS and 1% penicillin and streptomycin, were used. The cell lines were transfected with 0.5 μg/μl pcDNA3.1-CG4928-Flag (Invitrogen, 17AD6RSP), pcDNA3.1-CG4928-eGFP (Invitrogen, 17ADONXP) (for sequences, see Supplementary Dataset S3), or pcDNA3.1-empty (Invitrogen) plasmids using Lipofectamine 3000 (Thermo Scientific) and Fugene HD transfection reagent [Promega, membrane preparation and solid-supported membrane (SSM)-based electrophysiology].

#### Immunocytochemistry

Immunocytochemistry (ICC) was performed as described in Perland et al. (2016) on transfected SH-SY5Y cells and wild-type HEK293T cells (only anti-UNC93A) with antibodies all diluted in anti-UNC93A [rabbit, 1:100ab69443, Abcam, (Ceder et al., 2017)], anti-KCNK3 [mouse, 1:100, ab186352 Abcam, (Schmidt et al., 2015)], anti-KCNK9 (mouse, 1:100, Sigma, K0514), and anti-Flag [mouse, 1:200, F3165, Sigma, (Sriramachandran et al., 2019)]. Images were acquired using Olympus microscope BX55 with an Olympus DP73 camera and the cellSens Dimension v1.14 (Olympus) and were then handled using ImageJ, Fiji edition (Schindelin et al., 2012). The epitope against UNC93A was pair-wise aligned against the three isoforms of CG4928 (FBpp0074118, FBpp0074119, and FBpp0309738) using EMBOSS Needle (global alignment) and EMBOSS Water (local alignment) to investigate protein identity and similarity (Li et al., 2015) (Supplementary Dataset S4).

#### Proximity Ligation Assay

Transfected and non-transfected SH-SY5Y cells were washed in 1 × PBS and fixed using 4% formaldehyde (Sigma Aldrich) for 30 min, followed by permeabilization with 0.1% Triton-100 (Sigma Aldrich) for 10 min. PLA was performed using the Duolink^TM^ *in situ* detection reagent orange kit (Sigma Aldrich) according to the manufacturer’s instructions. Briefly, the cells were washed twice in 1 × Tris-buffered saline with Tween-20 (TBST) before these were blocked in Duolink^TM^ blocking buffer for 1 h at room temperature, followed by incubation with primary antibodies anti-UNC93A, anti-KCNK3, anti-KCNK9, and anti-Flag overnight at 4°C. The cells were washed twice in 1 × TBST and incubated for 1 h at room temperature with PLA probes diluted 1:5. The cells were once again washed before incubation in hybridization solution (diluted 1:5) for 15 min at 37°C. Ligation (ligation solution diluted 1:5, ligase diluted 1:40) was performed at 37°C for 15 min before amplification (amplification stock diluted 1:5, polymerase diluted 1:80) at 37°C for 90 min. The cells were washed twice for 10 min in 1 × Wash Buffer B at room temperature before washing twice for 2 min in TBST and detection solution. The detection solution (diluted 1:5) was added to the cells, followed by incubation for 30 min at 37°C. The cells were mounted with ProLong Gold antifade reagent with DAPI (Thermo Fisher), followed by imaging. Images were acquired with an Olympus microscope BX55 with an Olympus DP73 camera and the cellSens Dimension v1.14 (Olympus). The images were then handled using ImageJ, Fiji edition (Schindelin et al., 2012), and Duolink^TM^ Image tool (Olink Bioscience) was used to perform the analysis. GraphPad Prism, version 5, was used to retrieve graphs with mean (±SD) and perform the statistics. Kruskal–Wallis test, with Mann–Whitney as *post hoc* test, was used to detect differences in PLA signal (adjusted *p*-values: ^∗^*p* < 0.0491, ^∗∗^*p* < 0.00995, ^∗∗∗^*p* < 0.00099).

#### Membrane Preparation

The transfected HEK293T cells were collected in 1 × PBS using a cell scraper. The cells were lysed in lysis buffer containing 250 mM sucrose in 10 mM Tris, pH 7.5, and protease inhibitor cocktail using a freeze/thaw method. The lysates were centrifuged at 6,000 × *g* at 4°C for 10 min to isolate the nuclear pellet. Later, the supernatant was centrifuged at 100,000 × *g* at 4°C for 30 min to isolate the membrane pellet. This membrane pellet was dissolved in storing buffer containing 140 mM NaCl and 2 mM MgCl2 in 30 mM Tris, pH 7.5. The isolated membrane samples were then used for western blot and further analyzed by Nanion Technologies (Munich, Germany) through SSM-based electrophysiology to identify possible substrates.

#### Western Blot

Western blot was performed as described in Perland et al. (2016). The protein concentration of membrane samples from HEK293T cell cultures were analyzed using tryptophan measurement on a Tecan plate reader. Lysis buffer (25 mM Tris pH 7.8, 150 mM NaCl, 1 mM EDTA, 5% glycerol, 1% Triton X-100, and protease inhibitor cocktail) was added to the membrane samples before denaturation using dithiothreitol and sodium dodecyl sulfate containing Laemmli sample buffer at 95°C for 5 min. Then, 5 μg was added, and as reference, a molecular weight marker was used (prestained dual color, Bio-Rad). Anti-UNC93A (1:100) was used for protein visualization. The membrane was developed using Clarity Western ECL Substrate (Bio-Rad), and the staining was visualized using a CCD camera (Bio-Rad). The staining was compared with the molecular weight marker using Image Lab software v5.2.1 build 11 (Bio-Rad). Western blot was quantified using ImageJ, Fiji edition (Schindelin et al., 2012), and protein expression was normalized against β-actin. GraphPad Prism, version 5, was used to compile graphs.

#### SSM-Based Electrophysiology

Solid-supported membrane-based electrophysiology using SURFE^2^RN1 was performed as described in Bazzone et al. (2017) on membrane samples obtained from transfected HEK293T cells. The membrane was run on three sensors per replicate: duplicates (control: empty vector-transfected cells) and triplicates (for remaining runs). In short, 200 mM fructose, galactose, glucose, lactose, saccharose, or xylose was added to the membranes after 1 s and removed after approximately 2 s. SGLT1 was used as a positive control, and 250, 100, 20, 4, and 1 mM glucose was added. For amino acids, 20 mM of glycine, glycylglycine, alanine, glutamate, arginine, lysine, leucine, proline, cysteine, glutamine, aspartate, and allantoin was added after 1 s and removed after 2 s. PepT1 was used as a positive control and 100, 33, 11, 3.7, 1.2, and 0.4 mM glycylglycine were added. Graphs were generated using GraphPad Prism, version 5.1, and current (nA) was plotted against time (s) for each compound.

#### Apoptosis, Cell Cytotoxicity, and Viability Measurement

Apoptosis, cell cytotoxicity, and viability measurements were performed on (I) transfected HEK293T cells 24 h post-transfection and on (II) 24-h post-transfected HEK293T cells kept on different media [control: DMEM (Thermo Scientific); no glucose: DMEM, no glucose, no glutamine, no phenol red (A1443001, Thermo Scientific); high glucose; no glucose, DMEM supplemented with 50 mM Glucose (Sigma Aldrich); high-NaCl DMEM: DMEM media supplemented with 10 g/L extra NaCl; high KCl: DMEM media supplemented with 0.4 g/L extra KCl; low-NaCl media: MEM vitamin solution (100×) (11120037, Thermo Scientific), MEM amino acids solution (50×) (11130051, Thermo Scientific), 0.4 g/L KCl, 0.264 g/L CaCl_2_, 0.2 MgSO_4_, and 25 mM glucose (Sigma Aldrich); low-KCL media: MEM vitamin solution (100×) (11120037, Thermo Scientific), MEM amino acid solution (50×) (11130051, Thermo Scientific), 3.7 g/L NaHCO_3_, 0.14 g/L NaH_2_PO_4_, 0.2 g/L KCl, 0.264 g/L CaCl_2_, 0.2 MgSO_4_, and 25 mM glucose (Sigma Aldrich)] using the ApoTox-Glo^TM^ Triplex kit (Promega) according to the manufacturer’s protocol. Fluorescence and luminescence were measured using a BMG Labtech Omega plate reader. The (I) mean fluorescence and luminescence signal (±SEM) (*n* = 2, with 12 measurement points in each) and the (II) mean fluorescence and luminescence signal (±standard error of the difference) (*n* = 6) were plotted using GraphPad Prism 5, and differences were studied using (I) unpaired *t*-test (^∗^*p* < 0.05, ^∗∗^*p* < 0.01, ^∗∗∗^*p* < 0.001) and (II) appropriate ANOVA [one-way ANOVA (differences in apoptosis) or Kruskal–Wallis (differences in cytotoxicity)] with unpaired *t*-test or Mann–Whitney as *post hoc* test (adjusted *p*-values: ^∗^<0.0489, ^∗∗^<0.0099, ^∗∗∗^<0.0001).

#### Membrane Potential Measurement

The membrane potential on transfected cells was measured using the FluoVolt^TM^ Membrane Potential kit (Ceriani and Mammano, 2013; Bedut et al., 2016) (Thermo Scientific) according to the manufacturer’s instructions. Briefly, cells were washed twice in Live Cell Imaging Solution (LCIS; Thermo), before 2 ml FluoVolt^TM^ loading solution was added, and incubated for 15 min at room temperature. The FluoVolt^TM^ loading solution was removed, and the cells were washed twice in LCIS. Then, 2 ml of 20 mM glucose stock in LCIS was added, followed by imaging using fluorescence signal which was recorded using an ImageXpress Micro XLS (Molecular Devices) microscope with ×20 objective. Images were taken at an interval of 10 s, for 10 timepoints, with FITC filter (excitation: 482 nm; emission: 536 nm). Each well was imaged for two sites in FITC channel with four replicates. All experiments were performed at a controlled temperature of 37°C. The basal membrane potential was measured as well as after treatment with valinomycin (V1644, Thermo Scientific). The images were handled, and the fluorescent intensity was measured using ImageJ, Fiji edition (Schindelin et al., 2012). The average fluorescent intensity per area was calculated from each frame at each timepoint per well. Graphs with 95% confidence interval were generated, and statistical analysis was performed using GraphPad Prism 5. Unpaired *t*-tests were performed (^∗^*p* < 0.05, ^∗∗^*p* < 0.01, ^∗∗∗^*p* < 0.001).

## Results

### CG4928 Is Evolutionary Conserved and Orthologs to UNC93A

**FIGURE 1:**
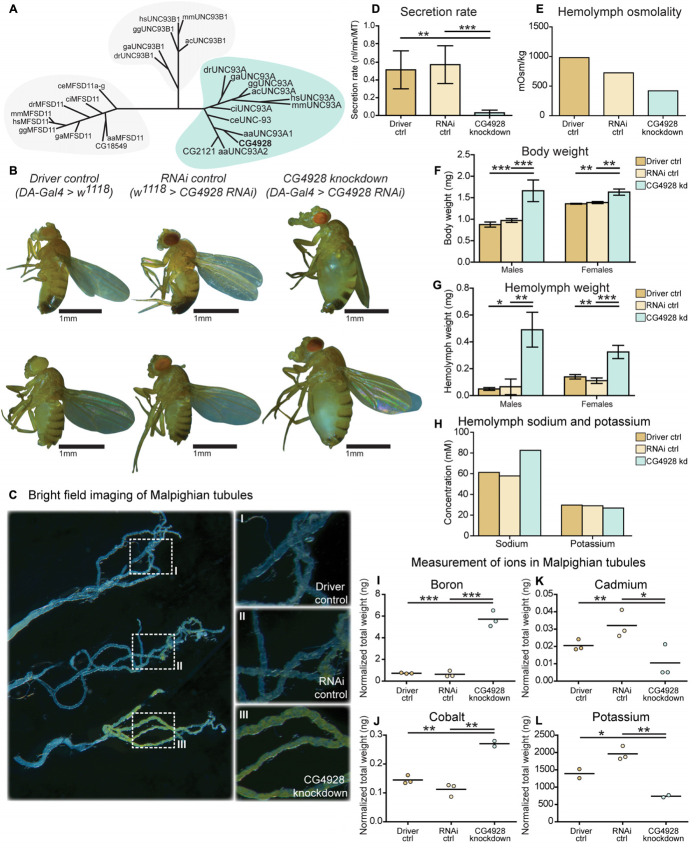
CG4928 is evolutionary conserved and crucial for normal renal function in *D. melanogaster*. Related proteins to UNC93A were searched for using a hidden Markov model. **(A)** UNC93A had orthologs in *A. aegypti*, *A. carolinensis*, *C. elegans*, *C. intestinalis*, *D.* rerio, *D. melanogaster*, *G. aculeatus*, *G. gallus*, and *M. musculus*. Closest relatives were UNC93B1 and MFSD11. *CG4928* was ubiquitously knocked down using *Da-Gal4*. **(B)** *CG4928* knockdown males and females developed edema in 28°C compared with Driver control and RNAi control. Bright-field images displaying the Malpighian tubules obtained from Driver control, RNAi control, and *CG4928* knockdown females were captured where **(C)** *CG4928* knockdowns showed an accumulation of yellow-colored pigments in the Malpighian tubules, close-up **(C)** I–III. Ramsay assay was performed on Malpighian tubules from adult females (*n* = 50). Secretion rate was measured after 2 h of secretion, average secretion rate (±SD) was plotted, and differences were calculated using one-way ANOVA with Bonferroni’s corrections (*p < 0.05, **p < 0.01, ***p < 0.001). **(D)** *CG4928* knockdown flies had a lower secretion rate compared with controls. Body and hemolymph samples were collected to measure osmolality, differences in body and hemolymph weight, and concentrations of sodium and potassium. Then, 20 μl of hemolymph sample from adult flies (*CG4928* knockdown *n* = 500, Driver control *n* = 800, RNAi control *n* = 800) was collected to measure osmolality and sodium and potassium concentrations. **(E)** *CG4928* knockdowns had lower osmolality. Body and hemolymph weight were measured in males and females (*n* = 30); weight was calculated against the number of flies. Graphs represent mean (±SD); differences were calculated with Kruskal–Wallis and Dunn’s correction (*p < 0.05, **p > 0.01, ***p > 0.001). *CG4928* knockdown flies were heavier, **(F)** body and **(G)** hemolymph weight, than controls and were found to have **(H)** 38.7% higher concentration of sodium and 8.6% lower concentration of potassium. Ion levels in Malpighian tubules from adult females were measured using ICP-SFMS (*n* = 2–3 per genotype, with Malpighian tubules from 60 flies in each). Data are plotted with a scatter plot; differences were calculated using unpaired *t*-test with Bonferroni’s correction (adjusted *p*-values: *p < 0.0492, **p < 0.0099, ***p < 0.0001). *CG4928* knockdown flies had altered levels of **(I)** boron, **(J)** cadmium, **(K)** cobalt, and **(L)** potassium.

Previously, hsUNC93A has been presented to be conserved in mammals and *C. elegans*, but the proteome from *D. melanogaster* has not been included. Here a Hidden Markov model for UNC93A was built and used to search for related sequences. Nine proteomes were included in the search: *A. aegypti*, *Anolis carolinensis*, *C. elegans*, *Ciona intestinalis*, *Danio rerio*, *D. melanogaster*, *Gasterosteus aculeatus*, *Gallus gallus*, and *M. musculus*. Orthologs to UNC93A were found in all included proteomes representing several branches of organisms (Table 1), and its closest relatives MFSD11 and UNC93B1 were also identified, consistent with earlier findings (Ceder et al., 2017). UNC93A had one ortholog in all species except *D. melanogaster* (CG4928, 34.8%; CG2121, 26.8%) and *A. aegypti* (aaUNC93A1, 30.2%; aaUNC93A2, 37.1%), where two orthologs were found. MFSD11 was found in all proteomes except in *A. carolinensis*, and UNC93B1 was not found in *D. melanogaster*, *A. aegypti*, and *A. carolinensis* (Figure 1A). Furthermore, global protein alignments between human proteins and orthologs in *D. melanogaster* were studied using EMBOSS Needle (Li et al., 2015). CG4928 was found to be most identical to hsUNC93A, indicating that CG4928 is the ortholog to UNC93A. However, of the two copies identified in *D. melanogaster*, CG4928 also had the highest resemblance to hsUNC93B1, but the protein identity was lower between these two orthologs compared with the protein identity of CG4928 and hsUNC93A. CG18549 is the orthologous protein to hsMFSD11 (Table 2).

**TABLE 1:** Amino acid identity comparison (Li et al., 2015; Madeira et al., 2019) of human protein sequences of UNC93A, UNC93B1, and MFSD11 and their identified orthologs.

Species	Protein name and amino acid identity		

*H. sapiens*	UNC93A	UNC93B1	MFSD11
*A. aegypti*	aaUNC93A1 30.2%		aaMFSD11 44.9%
	aaUNC93A2 37.1%		
*A. carolinensis*	acUNC93A 57.3%	acUNC93B1 66.2%	
*C. elegans*	ceUNC-93 20.6%		ceMFSD11g 32.5%
*C. intestinalis*	ciUNC93A 39.6%		ciMFSD11 50.4%
*D. melanogaster*	CG4928 34.8%		CG18549 41.5%
	CG2121 26.8%		
*D. rerio*	drUNC93A 55.2%	drUNC93B1 56%	drMFSD11 66.7%
*G. aculeatus*	gaUNC93A 55.9%	gaUNC93B1 61%	gaMFSD11 64.6%
*G. gallus*	ggUNC93A 64.1%	ggUNC93B1 57.4%	ggMFSD11 81.8%
*M. musculus*	mmUNC93A 71.9%	mmUNC93B1 87.4%	mmMFSD11 93.1%

**TABLE 2:** Comparing the amino acid identity of UNC93 protein sequences identified in human, *D. melanogaster*, and *C. elegans*.

	hsUNC93A (%)	hsUNC93B1 (%)	ceUNC-93 (%)
CG4928	34.8	20.9	22
CG2121	26.8	16. 8	21.3

*hs, *H. sapiens*; ce, *C. elegans*.*

### *CG4928* Is Expressed in Malpighian Tubules

The gene expression of *CG4928* in Malpighian tubules and in the intestine was investigated using the GAL4-UAS system coupled to GFP. The expression was visualized by crossing the enhancer trap line, *CG4928-Gal4*, to the UAS line coupled to GFP, and the organ was dissected and imaged. Expression was found in Malpighian tubules, located to round cells, compared with controls (*CG4928-Gal4* and *UAS-GFP* lines) (see Supplementary Figure S1A). Meanwhile, the expression in the intestine was difficult to image since food caused autofluorescence. However, a few cells were found to display GFP expression, but whether the cells are located within the wall of the intestine or are surrounding hemocytes remains uncertain (Supplementary Figure S1B).

### Ubiquitous Knockdown of *CG4928* Induce Renal Dysfunction

To study the function of *CG4928*, gene expression was reduced with the ubiquitous daughterless-Gal4 (*da-Gal4*) and *CG4928-UAS-RNAi* lines. Crosses were placed in 25°C, and offspring were raised in 28°C. Knockdown of *CG4928*, using the *CG4928-UAS-RNAi KK* line (*CG4928* knockdown), caused edema, where accumulation of fluid appeared both in the body (abdominal cavity) and the Malpighian tubules in males and females raised at 28°C (Figure 1B), while offspring did not survive pupation at 29°C. The *CG4928-UAS-RNAi GD* line did not produce viable offspring. It was possible to get female offspring to enclose at 18°C and few of them developed edema (data not shown), but the number of offspring was limited, and knockdown was not sufficient (data not shown); hence, the line was not used further. To verify the knockdown of *CG4928*, qRT-PCR was performed. *CG4928* expression was reduced compared with the controls *da-Gal4 > w1118* (Driver control) and *w1118 > CG4928-UAS-RNAi KK* (RNAi control) (Supplementary Figure S1C). Due to the phenotype, *CG4928* knockdown was performed using several Malpighian tubule-specific Gal4 lines, but these crosses did not reproduce edema and were, hence, not used further. It could be that these are weaker compared to *da-Gal4* or that the expression needs to be reduced in all tissues that express *CG4928* for the phenotype to develop. There is one reported off-target gene *CG9122* that encodes a tryptophan hydroxylase, which was accounted for. When performing knockdown of the off-target gene, no edema was observed (data not shown).

Malpighian tubules were dissected to compare the morphology between the Driver control, RNAi control, and *CG4928* knockdown flies. The tubules collected from *CG4928* knockdown females had a prominent yellow-colored substrate and fluid accumulation (Figure 1C), observed within the cells, compared with both controls. To investigate the renal function in *CG4928* knockdown flies, Ramsay assay was performed. Briefly, the Malpighian tubules from females were dissected, and the secretion capacity was measured after 2 h of secretion. *CG4928* knockdown was found to result in a marked reduction of fluid secretion with a secretion rate five times lower than both controls (Figure 1D). To further investigate how the edema affected the osmotic balance, the hemolymph osmolality was measured. The Driver control had an osmolality of around 1,000 mOsm/kg, RNAi control had 800 mOsm/kg, and *CG4928* knockdown had 500 mOsm/kg (Figure 1E), indicating that the *CG4928* knockdown has a lower concentration of substances dissolved in the hemolymph. It is important to note that the osmolality for the control flies is higher than normal, probably due to desiccation when raised at high temperatures (Albers and Bradley, 2004).

Edema was quantified through body and hemolymph weighing, where both *CG4928* knockdown males and females were found to be heavier than controls (Figure 1F). The extracted hemolymph from both *CG4928* knockdown males and females weighed more compared with those of the controls (Figure 1G). Furthermore, the sodium and the potassium levels in the extracted hemolymph were measured. The sodium level in the 20-μl hemolymph extracted from *CG4928* knockdown was 38.7% higher, while the potassium level was 8.6% lower, compared with those of the controls (Figure 1H). However, the sample for the *CG4928* knockdown flies was collected from 500 flies and for both controls from 800 flies. This skewness in sample size would indicate that the masses of sodium and potassium were approximately 121 and 46% higher, respectively, in the *CG4928* knockdown flies compared with the controls.

### *CG4928* Knockdown Affect Ion Levels in Malpighian Tubules

Within the Malpighian tubules, there is a continuous flow of ions to drive the transport of various molecules. The accumulation observed in the abdominal cavity and the Malpighian tubules, the low secretion rate, and the altered status of the hemolymph indicate that there is a disturbance in cellular processes affecting the concentrations of molecules over the membranes of the cells in Malpighian tubules, but plausible also with other excretory organs such as the hindgut. However, this organ was not further examined.

To get a more detailed view, Malpighian tubules were dissected and an elemental (ion) analysis was performed. In total, 69 elements were measured using ICP-SFMS. *CG4928* knockdown flies had altered amounts of boron, cadmium, cobalt, and potassium (Figures 1I–L), where boron and cobalt were higher and cadmium and potassium were lower compared with those of the controls. For the remaining element, no alterations were observed or the detected levels were too low (Supplementary Table S1).

### CG4928 Has 12 Transmembrane Helices Predicted to Fold Like MFS Transporters

**FIGURE 2:**
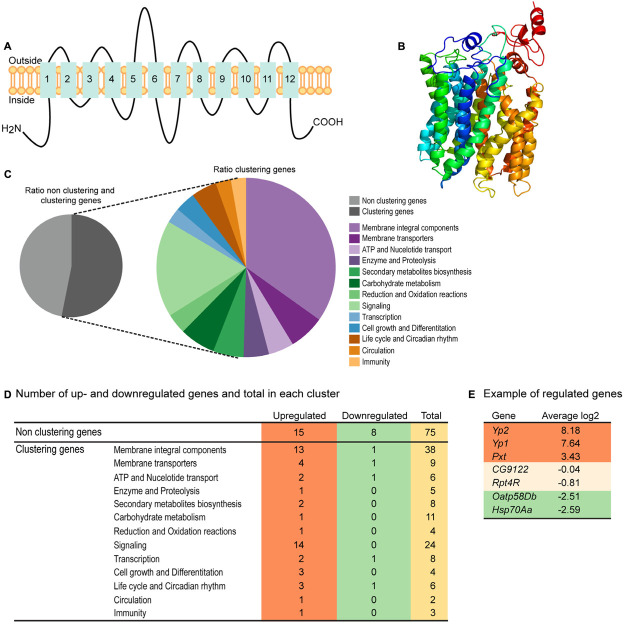
CG4928 has similar protein folding with the major facilitator superfamily (MFS) proteins, and reduction in *CG4928* expression affects membrane-bound biosynthesis and signaling genes. Homology modeling of CG4928 was performed using Protter and Phyre2 (Omasits et al., 2014; Kelley et al., 2015). The secondary structure of **(A)** CG4928 revealed 12 transmembrane helices. The tertiary structure illustrates a globular protein **(B)** with helices packed similar with the other MFS proteins (Yan, 2015). The rainbow gradient illustrates helices from N-termini (dark blue) to C-termini (red). Ion total RNA sequencing was performed on Driver control, RNAi control, and *CG4928* knockdown flies. The result was analyzed using STAR mapper and Cufflink software. **(C)** Pie chart illustrating the ratio between clustering and non-clustering genes; 53.1% of the genes could be divided into 13 clusters according to DAVID functional annotation bioinformatics microarray analysis (Huang et al., 2007); some genes were affiliated to several clusters. In total, 160 genes were found: 31.3% upregulated and 7.5% downregulated. **(D)** Most of the upregulated genes were found among the non-clustering genes (15 genes), the genes belonging to the membrane integral components (13 genes), and the signaling (14 genes) clusters. Downregulated genes were found in membrane integral components, membrane transporters, ATP and nucleotide transport, and transcription and life cycle and Circadian rhythm clusters. Examples of genes found in the analysis are **(E)** upregulated—*YP2*, *YP1*, and *Pxt*, non-altered—*CG9122* (off-target gene for *CG4928*) and *RPT4R*, and downregulated—*Oatp58Db* and *Hsp70Aa*.

Homology modeling using PROTTER (Omasits et al., 2014) predicted a 2D topology of CG4928 (Figure 2A) similar to the common structure of MFS proteins with 12 transmembrane helices (Madej et al., 2013). The homology model for CG4928 has long N- and C-termini that are present on the same side of the membrane compared to the predicted model for hsUNC93A that has shorter termini (Ceder et al., 2017). The tertiary model predicted using Phyre2 (Kelley et al., 2015) revealed a globular protein that folds like MFS proteins (Yan, 2015) (Figure 2B). Furthermore, 89% of the amino acids were modeled with 99.9% confidence, and 12% of the amino acids matched the template structure. The high confidence of the overall assimilation suggests that CG4928 has the overall structure illustrated.

### RNA Sequencing Reveals *CG4928* Knockdown to Affect Membrane-Bound Biosynthesis and Signaling Genes

RNA sequencing was performed on *CG4928* knockdown flies and controls to investigate if *CG4928* knockdown caused alterations in the mRNA expression profile of the flies. The sequencing was performed using Ion Total RNA sequencing, and the results were analyzed using STAR mapper (Dobin et al., 2013) and Cufflink (Trapnell et al., 2010, 2013) software. Genes were considered differentially expressed if they were significantly different (log2 fold change > 1.5 or <−1.5) in *CG4928* knockdown flies compared with both controls. This approach generated 160 differentially expressed genes, and of those, approximately 53.1% (62 genes) could be categorized into 13 gene clusters: (i) integral membrane components, (ii) membrane transporters, (iii) ATP and nucleotide transport, (iv) enzyme and proteolysis, (v) secondary metabolites biosynthesis, (vi) carbohydrate metabolism, (vii) reduction and oxidation reactions, (viii) signaling, (ix) transcription, (x) cell growth and differentiation, (xi) life cycle and circadian rhythm, (xii) circulation, and (xiii) immunity (Figure 2C). It is noteworthy that one gene can cluster into more than one gene cluster. In total, 50 genes were upregulated and 12 were downregulated (Figure 2D and Supplementary Table S2), where one third of the regulated proteins, mostly orphan genes without a known function, were categorized as non-clustering genes. The emerging pattern did not point to a clear pathway that *CG4928* could be involved in; however, it appears important for normal cellular status since genes responsible for transcription, membrane permeability, transport, and synthesis were affected. Some regulated and non-regulated genes were *Yp1*, *Yp2*, and *Pxt* that were upregulated, *Oatp58Db* and *Hsp70Aa* that were downregulated, and *CG9122* (off-target to *CG4928*) and *Rpt4R* that were not altered (Figure 2E). To verify RNA sequencing, qPCR was performed on matched samples for four of the genes: *Pxt*, *CG9122*, *Rpt4R*, and *Oatp58Db* (Supplementary Figures S1D–G). Gene expression was similar compared with the findings from RNA sequencing, except *Oatp58Db* that was upregulated when performing qPCR.

### *CG4928* Knockdown Flies Are Sensitive to Starvation

Since *CG4928* knockdown implies to affect the ion homeostasis and genes involved in metabolism, the metabolic condition of the *CG4928* knockdown flies was studied further. We explored several aspects of metabolism, starvation tolerance, and locomotor by monitoring their activity in DAMS and food intake as well as measuring the stored and circulating macronutrient levels. The *CG4928* knockdowns had lower starvation tolerance, but no difference in activity and food intake were observed (Supplementary Figures S1H–J). However, differences in stored and circulating sugars, fats, and proteins could give an indication why they starve faster. The *CG4928* knockdowns had higher levels of stored and circulating sugars before starvation (0 h) (Supplementary Figures S1K–N). No difference was observed after 12 and 24 h of starvation. The levels of sugars gradually decreased over the time of starvation, suggesting that *CG4928* knockdown flies can recruit sugars and do not deplete the storage too quickly. A similar result was observed for circulating lipids (Supplementary Figure S1O). No difference was seen for the protein levels in the hemolymph (Supplementary Figure S1P). Hence, the sensitivity to starvation could not be explained by altered activity, food intake, and macronutrient deficiencies; hence, what exactly provokes this phenotype is still unknown.

### The Edema Is Minimized With Amiloride and Rescued With Ion-Free Diets

Five-day-old *CG4928* knockdown male flies and controls were transferred and fed diuretics commonly used for treating edema in humans and ion-free (containing sugar and yeast) diets to treat the edema. In addition, diets enriched with NaCl or KCl were used. Photos of the flies were captured, and body and hemolymph weight was measured, respectively.

**FIGURE 3:**
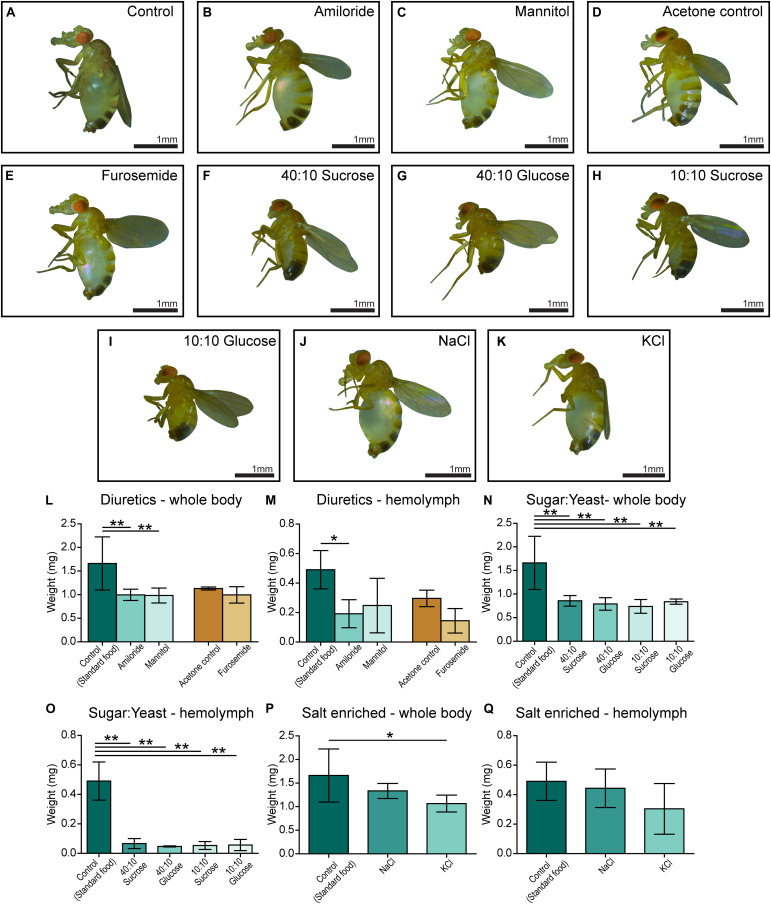
Amiloride minimizes edema and ion-free diets rescue the edema. *CG4928* knockdown flies were kept on diuretic sugar/yeast (ion-free) and salt-enriched diets. All diets are compared with the standard food control except furosemide that is compared with an acetone control. Images were captured of flies on each diet: **(A)** control (standard food), **(B)** amiloride, **(C)** mannitol, **(D)** acetone control, **(E)** furosemide, **(F)** 40:10 sucrose, **(G)** 40:10 glucose, **(H)** 10:10 sucrose, **(I)** 10:10 glucose, **(J)** NaCl-enriched food, and **(K)** KCl-enriched food. Body and hemolymph weight were measured (*n* = 30), and weight was normalized against the number of flies. Graphs represent mean (±SD); differences were calculated with Kruskal–Wallis and Dunn’s correction (*p < 0.05, **p > 0.01, ***p > 0.001). **(L)** The body weight of *CG4928* knockdown flies was reduced with amiloride and mannitol, but not furosemide. **(M)** Hemolymph weight was reduced with amiloride. All sugar/yeast (ion-free) diets reduced the **(N)** body weight and **(O)** hemolymph weight. **(P,Q)** *CG4928* knockdowns fed NaCl-enriched food did not affect the edema, while those fed with KCl-enriched food weighed less than flies that were raised on standard food.

Amiloride—a blocker for epithelial sodium channels (ENaCs), mannitol—a sugar alcohol, and furosemide—an inhibitor of the Na-K-Cl cotransporter (NKCC) were used. Diuretics were dissolved in water and added to the standard food, except furosemide that was dissolved in acetone before its addition to food. On standard food, the *CG4928* knockdowns (used as control) developed edema as observed before (Figure 3A). Amiloride reduced both body and hemolymph weight compared with *CG4928* knockdown flies on standard food (Figures 3B,L,M), while mannitol only decreased the body weight (Figures 3C,L,M). Since furosemide was solved in acetone, the flies maintained on standard food with acetone (acetone control) were used as controls. The flies kept on food supplemented with acetone had a similar phenotype as the *CG4928* knockdown on standard food (Figures 3D,L,M), and furosemide did not rescue the phenotype (Figures 3E,L,M). When comparing the body and hemolymph weight against the Driver control and the RNAi control, only mannitol restored the body weight of *CG4928* knockdown flies compared with both controls, while the hemolymph weight was not affected by any of the diuretics (Supplementary Figures 1Q,T).

The ion-free diets were made with 0.1 mg/ml yeast and either sucrose or glucose in two concentrations, 0.1 and 0.4 mg/ml. Sugar and yeast were dissolved in deionized water. All diets restored the body and hemolymph weight and rescued the phenotype, both compared with the *CG4928* knockdown flies fed standard food (Figures 3F–I,N,O) and compared with the Driver and RNAi controls (Supplementary Figures S1R,U).

When feeding flies with sodium- and potassium-enriched food, the phenotype was not affected (Figures 3J,K,P,Q and Supplementary Figures S1S,V).

### UNC93A as a Regulatory Subunit of TASK^1^ Channels

**FIGURE 4:**
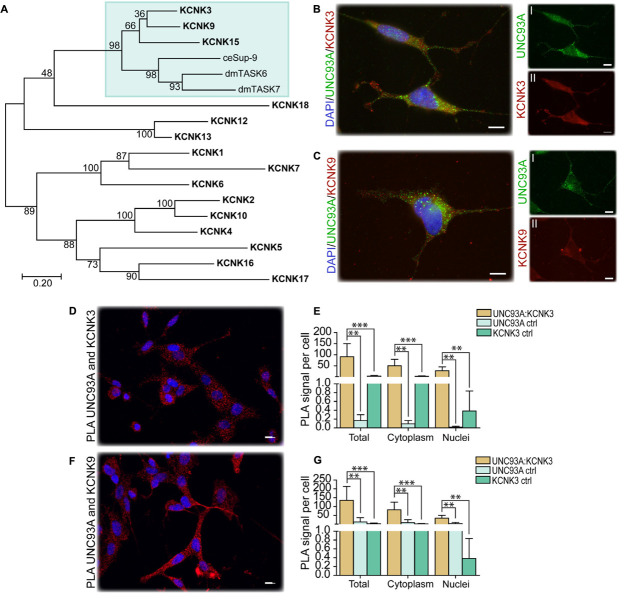
CG4928 as a regulatory protein of Twik-related acid-sensitive K^+^ 1 (TASK^1^) channels. The phylogenetic relationship between *C. elegans* (Sup-9), *D. melanogaster* (TASK6 and TASK7), and *H. sapiens* (KCNK proteins) was investigated. **(A)** Human KCNK3, KCNK9, and KCNK15 together with TASK6 and TASK7 in *D. melanogaster* were identified as closest relatives to Sup-9 in *C. elegans*. Protein localization was stained for using immunocytochemistry on wild-type SH-SY5Y cells for anti-UNC93A (green), anti-KCNK3 and anti-KCNK9 (red), and nucleus marker DAPI (blue). **(B)** UNC93A and KCNK3 staining was found in the same cells; the same was observed for **(C)** UNC93A and KCNK9. Co-localization was studied using proximity ligation assay (PLA) on wild-type SH-SY5Y cells, and the result was analyzed using Duolink^TM^ imaging tool. The PLA signal per cell was calculated for total, cytoplasm, and nuclei signals. The graphs display the average PLA signal per cell (±SD). Differences were calculated using Kruskal–Wallis with Bonferroni multiple-corrected Mann–Whitney as a *post hoc* test (*p < 0.049, **p < 0.0099, ***p < 0.001). Co-localization was found for **(D)** UNC93A and KCNK3, and the PLA signals observed in total, cytoplasm, and nuclei were higher compared with controls for **(E)** UNC93A and KCNK3. Similar results were observed for **(F**,**G)** UNC93A and KCNK9.

In *C. elegans*, Unc-93 is suggested to act as a regulatory subunit of Sup-9, an ortholog to the human KCNK family of potassium transports important for resting membrane potentials (Goldstein et al., 2001; Talley et al., 2001). The phylogenetic relationship between human, fly, and *C. elegans* was analyzed, and Sup-9 was found to be most similar to KCNK3, KCNK9, and KCNK15 in humans and TASK6 and TASK7 in flies (Figure 4A), which is consistent with earlier findings (de la Cruz et al., 2003). To investigate if KCNK3, KCNK9, and UNC93A co-localize, ICC and PLA were performed on wild-type and CG4928:Flag-transfected SH-SY5Y cells. KCNK15 was not included in the study since the antibodies that are commercially available were not suitable to use with the anti-UNC93A antibody. The immunoreactivity revealed similar staining patterns for UNC93A, KCNK3, and KCNK9 in wild-type SH-SY5Y cells (Figures 4B,C), and no signal was detected in the negative control (Supplementary Figure S2A). The immunoreactivity for anti-Flag on CG4928:Flag-transfected SH-SY5Y cells also displayed an overlap with KCNK3 and KCNK9, while the negative control was blank (Supplementary Figures S2B–D). The ICC suggests that they are located in the same cell, and therefore PLA was conducted: the total, cytoplasmic, and nucleic PLA signal per cell was calculated using Duolink^TM^ Image tool. UNC93A and KCNK3 were found to co-localize (Figures 4D,E), and a comparable result was observed for UNC93A and KCNK9 (Figures 4F,G). In CG4928:Flag-transfected cells, anti-Flag, anti-UNC93A, and anti-KCNK3 were found to co-localize (Supplementary Figures S2E,F,M,N), while no or weak co-localization could be detected with KCNK9 (Supplementary Figures S2G,O). No PLA signal was detected in the negative controls for Flag, UNC93A, and KCNK3 antibodies, while weak signals were detected for KCNK9 (Supplementary Figures S2H–K).

### CG4928 as a Transporter

Human UNC93A has the characteristics and 2D topology to be an authentic transporter (Ceder et al., 2017), and it has been predicted to be a putative SLC (Perland and Fredriksson, 2017; Perland et al., 2017a). Hence, one can assume that CG4928 possesses similar characteristics, and the substrate profile of CG4928 was investigated. Before performing transport assays, we investigated the endogenous UNC93A expression in HEK293T cells using ICC to determine the baseline expression of UNC93A. Immunoreactivity for UNC93A was detected in the cells (Supplementary Figure S2L).

**FIGURE 5:**
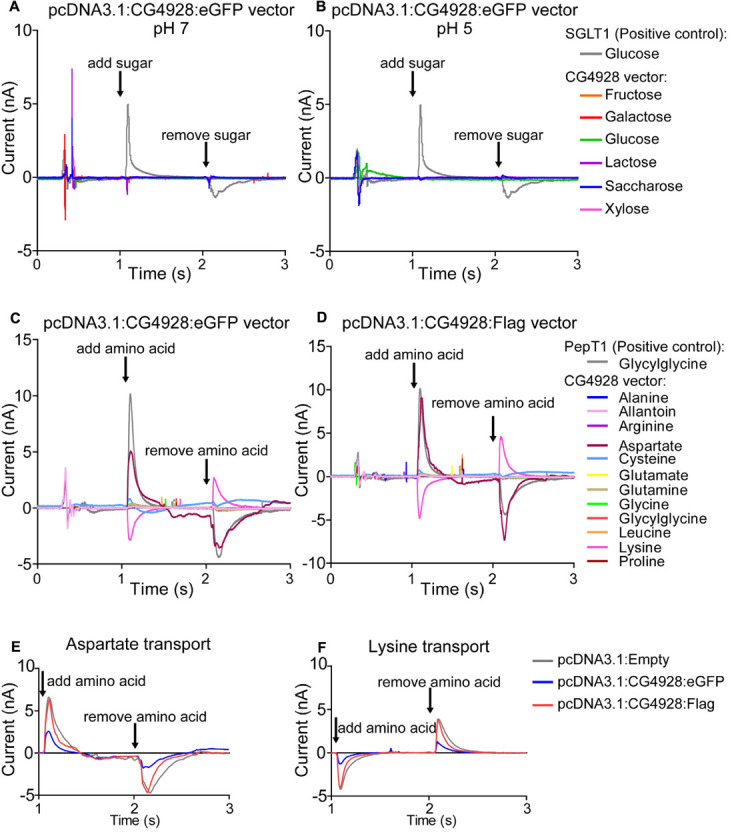
CG4928 as a transporter. To further study the function of CG4928, HEK293T cells were transfected with CG4928:eGFP, CG4928:Flag, and control (empty) vectors. The substrate profile was investigated on isolated membranes expressing the vectors using SSM-based electrophysiology (Bazzone et al., 2017). Six different sugars and 12 amino acids and peptides were tested. SGLT1 and PepT1 were used as positive controls. CG4928:eGFP-transfected cells did not transport any sugars at **(A)** pH 7 or **(B)** pH 5. Both **(C)** CG4928:eGFP- and **(D)** CG4928:Flag-transfected cells had altered currents for aspartate and lysine. However, when comparing the alteration in current to the empty vector, no differences were observed for **(E)** aspartate or **(F)** lysine.

Thereafter, HEK293T cells were transfected with CG4928:eGFP, CG4928:Flag, or control (empty) vectors, the membranes were isolated, and western blot was used to verify the transfection. There is no antibody available for CG4928, and therefore the human anti-UNC93A antibody was used during western blot. To determine the binding probability and specificity to CG4928 using the human anti-UNC93A, antibody protein alignments were performed. However, the epitope for anti-UNC93A is not specified by the company, except that it is an 18-amino-acid-long sequence located at the C-terminus of the human UNC93A. Therefore, protein alignments, both global and local, were performed between the three isoforms of CG4928 and human UNC93A (Supplementary Dataset S4). The alignment suggests that the antibody could bind to the fly ortholog, but probably not as specific as to the human protein. Anti-β-actin was used for normalization, and anti-UNC93A was used to stain for vector insertion. One band was detected for β-actin at 43 kDa, and one band for UNC93A was detected at 46 kDa (Supplementary Figures S2P,Q), which is consistent with previous findings (Ceder et al., 2017). Similar bands were detected for CG4928:Flag-transfected cells (data not shown). After normalization with anti-β-actin, the protein expression of UNC93A was higher in both CG4928:eGFP- and CG4928:Flag-transfected cells compared with the control (Supplementary Figures S2R,S). Hence, we proceeded with the transfected cells and performed SSM-based electrophysiology (Bazzone et al., 2017). In total, six sugars (fructose, galactose, glucose, lactose, saccharose, and xylose; 200 mM) and 12 amino acids and peptides (alanine, allantoin, arginine, aspartate, cysteine, glutamate, glutamine, glycine, glycylglycine, leucine, lysine, and proline; 20 mM) were tested. No changes in current were observed when adding the different sugars at pH 7 and 5 (Figures 5A,B and Supplementary Figures S2T–W). For amino acids, alterations in current for aspartate and lysine were observed (Figures 5C,D). However, a change was also observed for the control vector (Supplementary Figure S2X), and when the currents between all three vectors were compared, no difference was detected (Figures 5E,F).

### Overexpression of CG4928 Induces Apoptosis and Alters the Membrane Potential

**FIGURE 6:**
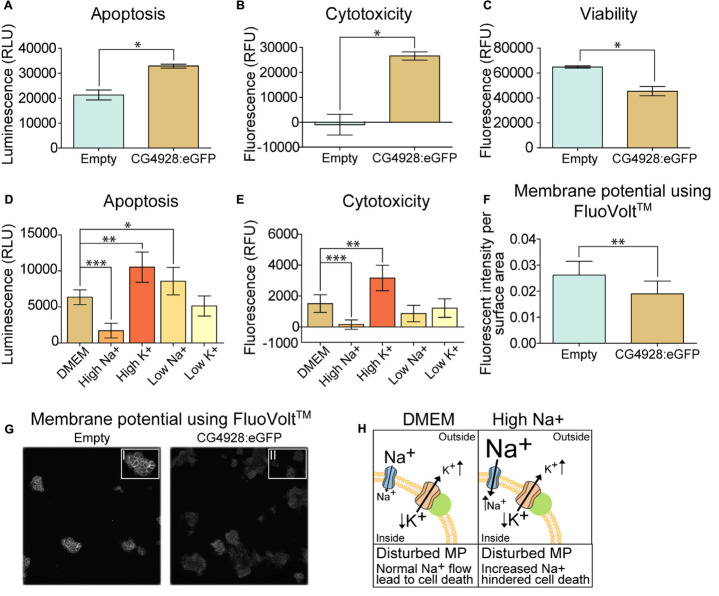
Overexpression of CG4928 induces apoptosis and alters the membrane potential. The CG4928:eGFP-transfected cells had a decline in cell number at 24 h post-transfection, and therefore apoptosis, cytotoxicity, and viability were monitored using the ApoTox-Glo^TM^ Triplex kit. Fluorescence and luminescence were measured, mean fluorescence and luminescence signals (±SEM) (*n* = 2 with 12 measured points in each) were plotted, and differences were studied using unpaired *t*-test (*p < 0.05, **p < 0.01, ***p < 0.001). CG4928:eGFP-transfected cells had **(A)** higher apoptosis, **(B)** cytotoxicity, and **(C)** lower viability. The CG4928:eGFP-transfected cells were maintained in different media [normal Dulbecco’s modified Eagle’s medium (DMEM), high sodium, high potassium, low sodium, and low potassium media] to investigate changes in cell death. The mean fluorescent and luminescence signals (±standard error of the difference) (*n* = 6) were plotted and CG4928:eGFP-transfected cells on normal DMEM media was used as control. Differences were calculated using appropriate ANOVA [one-way ANOVA (apoptosis) or Kruskal–Wallis (cytotoxicity)] with unpaired *t*-test or Mann–Whitney as *post hoc* test (adjusted *p*-values: *p < 0.0489, **p < 0.0099, ***p < 0.0001). **(D)** Changes in apoptosis were observed in transfected cells maintained on high sodium, high potassium, and low sodium, **(E)** while only high sodium and high potassium altered the cytotoxicity. The speculated interaction between CG4928 and TASK^1^ channels suggests that the results could be linked to the membrane potential. Therefore, the membrane potential was measured using the FluoVolt^TM^ membrane potential kit. The average fluorescent intensity per area was calculated from each frame at each timepoint per well (*n* = 4, images taken for two sites with an interval of 10 s for each of the 10 timepoints). Graphs with 95% confidence interval were generated and unpaired *t*-tests were performed (*p < 0.05, **p < 0.01, ***p < 0.001). **(F)** The basal membrane potential was lower in CG4928:eGFP-transfected cells compared with the control. **(G)** Representative image of the difference in fluorescent intensity between CG4928:eGFP-transfected cells and the controls. **(H)** Taken together, it can be speculated that CG4928 is important for TASK^1^ channel function and/or regulation. Overexpression of CG4928 probably caused potassium leakage, leading to disturbed membrane potential and cell death. Cell death could be hindered by culturing the CG4928-overexpressing cells in high sodium concentrations, but the mechanisms behind the rescue still remain uncertain.

An observation at 24 h post-transfection of CG4928 vectors was the increased cell death. Therefore, apoptosis, cytotoxicity, and viability were measured using the ApoTox-Glo^TM^ Triplex kit, where protease activity and caspase-3/7 cleavage are measured (Niles et al., 2007). The CG4928:eGFP-transfected cells had a higher degree of apoptosis and cytotoxicity and reduced viability compared with the control (Figures 6A–C). Similar results were obtained for HEK293T cells transfected with CG4928:Flag (data not shown). To further investigate if cell death could be prevented, CG4928:eGFP cells and control cells were maintained on different media: DMEM (control), high sodium, high potassium, low sodium, and low potassium. The average luminescence or fluorescence differences between CG4928:eGFP cells and control cells were investigated (Figure 6), and the comparisons between CG4928:eGFP cells and control cells for each media are plotted in Supplementary Figures S2Y,Z, where also no glucose and high glucose media were included. The degree of apoptosis and cytotoxicity decreased when cells were kept on high-sodium media, while the conditions were unchanged or worsened on high-potassium, low-sodium, and low-potassium media (Figures 6D,E). Compared with the control, only high sodium hindered apoptosis, while the increased cytotoxicity was only reproduced in cells maintained on DMEM and high-potassium and low-potassium media (Supplementary Figures S2Y,Z). The results show that overexpression of CG4928 induces cell death when maintained on media containing 150 mM sodium, but 300 mM of extracellular sodium helps the cell to survive. This could be due to a change in the net charge over the membrane if CG4928 forms a complex with TASK^1^ channels as suggested. If this is true, the CG4928-overexpressing cells should display a disturbance of the resting (basal) membrane potential. To test this, the potential was measured using FluoVolt^TM^ membrane potential kit, which detects changes in membrane potential (Ceriani and Mammano, 2013; Bedut et al., 2016). Briefly, CG4928:eGFP- and control-transfected cells were stained with FluoVolt^TM^, images were taken with FITC at an interval of 10 s for 10 timepoints in four wells per vector, and the average fluorescence intensity per area was calculated for each well. As a positive control, valinomycin was added to the transfected cells, which depolarized the cell and increased the fluorescence signal. Valinomycin, as predicted, increased the membrane potential (Supplementary Figure S2AA). The basal membrane potential was found to be lower in CG4928:eGFP-transfected cells compared with the control (Figures 6F,G), supporting the hypothesis that CG4928 alters membrane potential, probably by dysregulating KCNK3 and/or KCNK9 (Gabriel et al., 2012; Sun et al., 2016), leading to hyperpolarization of the cells (as illustrated in Figure 6H).

## Discussion

Lately, several orphan transporters suggested to be putative SLCs (Perland and Fredriksson, 2017; Perland et al., 2017a) have begun to be characterized with focus on evolutionary relationship, protein structure prediction, and expression profile (Perland et al., 2016, 2017b,c; Ceder et al., 2017; Lekholm et al., 2017). Previous phylogenetic analyses predicted UNC93A to be evolutionary preserved (Ceder et al., 2017; Perland and Fredriksson, 2017; Perland et al., 2017a), which we also confirmed. In comparison to mammals, two copies of UNC93A, CG4928, and CG2121 were identified. CG4928 was found to be most similar to hsUNC93A but also resembled human UNC93B1, indicating that this orthologous protein in *D. melanogaster* might possess features from both proteins in human. The tissue expression of the two UNC93 proteins varies in humans. According to NCBI^2^ and the human protein atlas^3^, UNC93B1 is abundantly expressed in the body, whereas UNC93A is mainly expressed in the digestive tract and the kidney (Fagerberg et al., 2014; Uhlen et al., 2015). However, *Unc93a* has been found to have an abundant gene expression in mice, but also here the expression was higher in peripheral organs, with a high expression reported in the intestine, liver, spleen, and fat tissues (Ceder et al., 2017). In *D. melanogaster*, little is known about the orthologs’ expression, and verifications have not been performed for all tissues. However, *CG4928* is reported to be expressed in several, but not all, organs of the fly, with highest expression in the salivary gland, hindgut, and Malpighian tubules. Meanwhile, in larvae, the expression is much more restricted to the gut and Malpighian tubules^4^ (Robinson et al., 2013). *CG2121*, on the other hand, is lowly to moderately expressed in almost all organs in the adult fly (Robinson et al., 2013). According to the tissue expression profiles in these three species, UNC93A and CG4928 seem to be more alike compared to UNC93B1 and CG4928.

*CG4928* is, according to transcriptome analyses, highly expressed in the digestive system and Malpighian tubules in both larvae and adult fly (Chintapalli et al., 2007). A study has found that *CG4928* is 13 times more highly expressed in Malpighian tubules compared to whole-body expression (Wang et al., 2004), which corresponds well to our findings. *CG4928* was found in large cells in the lower and the main segment of Malpighian tubules, which are simplified versions of the human kidneys in terms of their cellular structure and function (Gautam et al., 2017). Within the Malpighian tubules, there are two major cell types carrying out most actions, the principal cells and the stellate cells (Sozen et al., 1997). *CG4928* is possibly expressed in the principal cells based on the morphological similarities to these cells (Sozen et al., 1997; Soderberg et al., 2011; Roy et al., 2015), which are important for maintaining ion and fluid balance. GFP expression was also detected in cells surrounding, or possibly located within, the membrane of the midgut and the hindgut, but the number of cells expressing GFP, hence being CG4928-expressing cells, were few and did not correspond well to the microarray data. CG4928 has also been reported in hemocytes (Stofanko et al., 2008), the “white blood cells” of flies (Holz et al., 2003). When puncturing and mounting whole larvae from *Gal4-CG4928 > UAS-GFP* offspring to visualize the tissue expression, we detected GFP in “free-floating” cells, probably hemocytes, but we were not able to verify the results (data not shown). It is important to note that further research is needed to establish the role of CG4928 in hemocytes, the midgut, and the hindgut. However, it is possible that the observed phenotype is linked to one or more of these tissues.

Interestingly, in comparison to the protein structure of UNC93A, CG4928 is predicted to have longer N- and C-termini, which is more similar to the predicted structure of hsUNC93B1, once more suggesting that CG4928 might possess features from both variants in humans. Despite structural evidence of both UNC93A (Ceder et al., 2017) and CG4928 to be authentic transporters, we were not able to identify a substrate of transport. The setup of the SSM-based electrophysiology has its limitations and, for example, we cannot detect ion–ion cotransport nor uniport activity. Therefore, there is still the possibility that CG4928 is an authentic transporter. However, there are SLC families that lack the ability to act as transporters themselves and that have lost the ability to facilitate transport (Fotiadis et al., 2013; Abbott, 2017); however, this is less likely for CG4928. So far, our results support the current suggested theory that it is a regulatory unit of potassium channels (Levin and Horvitz, 1992; de la Cruz et al., 2003). Both the immunostaining and PLA confirm the co-localization of UNC93A and CG4928 with KCNK3 and KCNK9, respectively, which supports the theory. It is noteworthy that UNC93B1 also features a 2D topology of an authentic transporter, but it transfers Toll-like receptor (TLR) from the endoplasmic reticulum to the plasma membrane (Lee et al., 2013; Huh et al., 2014) and maintain TLR protein expression by acting as a trafficking chaperone (Pelka et al., 2018) and not by acting as a typical SLC. These suggest that UNC93A could be involved in the trafficking and/or the retention of the TASK^1^ channels to the plasma membrane. Several other potassium channels also need regulatory subunits that both regulate their function and location (Capera et al., 2019), and there are several other protein families, e.g., anchoring proteins and scaffolding proteins, needed for the correct membrane insertion of potassium channels (Capera et al., 2019). Recently, new interesting functions have been added to the SLCs, and there are evidences that they form complexes and interact with other membrane proteins to form “transceptors” (Jung et al., 2015; Rebsamen et al., 2015; Wang et al., 2015) and “chansporters” (Neverisky and Abbott, 2015; Abbott, 2017, 2020). These findings give rise to a new hypothesis regarding CG4928 that, in contrast of only being a subunit, it might form a chansporter complex with TASK^1^ channels. In fact, the type of interaction and the crosstalk between channels and transporters have been detailed and elegantly described for other potassium channels, e.g., the KCNQs (Abbott, 2020). However, the exact mechanisms and the functional interaction between CG4928 and TASK^1^ channels are still not clear, and further studies are needed to establish if they act as a chansporter complex. However, despite not knowing the exact mechanism of action, the results presented here establish that CG4928 is vital for the normal physiology of the body and cell. Potassium, most commonly found in intracellular compartments, is important for several processes occurring in the body, e.g., motility (de la Cruz et al., 2003), vesicle release, blood glucose regulation, renal function, but most importantly, it sets and maintains the membrane potential (Adler and Fraley, 1977; Palmer and Clegg, 2016). TASK^1^ channels are so-called leak channels which are important for resting potential (Talley and Bayliss, 2002; Plant et al., 2012) and are regulated by factors such as pH. KCNK3 is an outwardly rectifying channel (Duprat et al., 1997; Plant et al., 2012), while KCNK9 is a pH-dependent, voltage-insensitive background channel (Chapman et al., 2000; Vega-Saenz, de Miera et al., 2001; Plant et al., 2012), where both channels allow the movement of potassium depending on the electrochemical gradient.

The correct plasma level of potassium is essential for tuning the resting potential of cells. We found that the overexpression of CG4928 in HEK293T cells leads to a reduction in basal membrane potential. The result indicates that the cell is less depolarized; hence, the cell will need greater-than-“normal” stimuli to depolarize itself. The loss of function in membrane potential halters several essential properties of the cell (Abdul Kadir et al., 2018), which most likely cause an induction in apoptosis and cytotoxicity. Except for potassium, sodium influx is a main event of depolarization (Seyama and Irisawa, 1967). Interestingly, apoptosis and cytotoxicity were hindered when the cells were maintained on high-sodium media, implying that the CG4928-overexpressing cells are less depolarized. The normal extracellular sodium concentration cannot initiate a change in membrane potential, while maintaining the cells in a high extracellular concentration of sodium can enable them to prevent apoptosis.

When analyzing the hemolymph content, we established that *CG4928* knockdown flies had higher sodium and potassium levels in the hemolymph and a lower level of potassium within the Malpighian tubules. Potassium is of great significance for the function of Malpighian tubules and, in contrast to mammals, the primary urine production in flies is potassium dependent (Dow et al., 1998). Furthermore, the secretion rate was found to be five times lower in the *CG4928* knockdown flies’ Malpighian tubules compared with that of the controls. Similar observations have been made for other potassium channels, e.g., *Irk1* and *Irk2*, two channels responsible for transepithelial potassium fluxes in the principal cells (Wu et al., 2015). Taken together, these two findings are most likely the causes of edema. However, CG4928 is, according to microarray analysis, also expressed in the hindgut, an organ that is equally important for systemic water homeostasis (Luan et al., 2015). The hindgut has also been suggested to be sensitive to elevated intracellular concentrations of sodium and potassium during external hypertonic states (Huang et al., 2002). Hence, the organ is proposed to possess an osmoprotective role, where sodium and potassium, by an unknown function, are transported out of the cell, returning the cell volume and the intracellular ion concentration back to normal (Huang et al., 2002; Luan et al., 2015). This corroborates our findings about the increased levels of sodium and potassium in the extracellular fluid collected from adult flies suffering from edema. Moreover, knockdown of the sodium- and chloride-dependent neurotransmitter transporter family ortholog *Ine*, which is expressed in the basolateral epithelium of the hindgut, results in findings similar to those seen in *CG4928* knockdown flies, e.g., sensitivity to desiccation, altered hemolymph volume and total body water mass (Luan et al., 2015). However, in comparison to the Malpighian tubule for which sophisticated tools have been developed to study its function, the mechanisms of water conservation, absorption, and secretion of the hindgut are less elucidated in the adult fly (Luan et al., 2015). This, together with the low GFP expression (which would indicate the CG4928 expression) visualized in the hindgut, made it cumbersome to evaluate the function of CG4928 in this particular organ, and the focus remained on the Malpighian tubules. Also, hindgut-specific Gal4 lines were not able to reproduce the edema, suggesting that this organ is not the main factor causing the edema, but it can neither be ruled out and further research is needed to establish if CG4928 can be a potential gene in the regulation of water homeostasis through the hindgut.

The edema was minimized by amiloride, but not mannitol and furosemide. The differences in the mechanism of action are the most likely explanation to this finding. Amiloride binds and blocks ENaCs and thereby inhibits sodium reabsorption, leading to excretion of sodium and water from the body, while reducing potassium excretion (Wishart et al., 2018), similar effects have been observed in *D. melanogaster* by acting *via* the Na+/H+ exchangers (Giannakou and Dow, 2001; Zelle et al., 2013). It is therefore possible that the phenotype is minimized by inhibiting transepithelial sodium secretion and thereby increasing fluid secretion, but it is not enough to treat the edema. In humans, mannitol is metabolically inert; however, it can be metabolized in flies. When feeding higher concentrations of mannitol, it is likely that not all is metabolized and therefore it acts as a weak osmotic molecule within the digestive tract, increasing water excretion *via* feces. Meanwhile, furosemide causes sodium, potassium, and chloride loss in the urine, leading to lowered water reabsorption by inhibiting the NKCC (Wishart et al., 2018). However, in flies, the NKCC is located on the apical membrane, which means that inhibition of NKCC will lead to less potassium uptake into the Malpighian tubules (Rodan et al., 2012). Since the primary urine production of flies is potassium dependent, fluid secretion will decrease, and the flies will retain the edema. Ion-free diets, regardless of sugar type and concentration, rescued the edema. This suggests that *CG4928* knockdown flies can handle endogenous ion levels, indicating that there are other biochemical pathways that have the capability to use, store, and handle the faulty potassium fluxes (Seifter and Chang, 2017).

If the potassium transport is not working, as suggested, other ion levels will be affected, which could be a reason to the altered boron, cadmium, cobalt, and sodium levels observed in *CG4928* knockdown flies. TASK^1^ channels are not known to facilitate the movement of these four ions. However, the transport of, e.g., boron and sodium is dependent on both passive and active coupled transport, and the transport is sometimes linked directly to the level of potassium (Arroyo et al., 2013; Schnetkamp, 2013) or through other molecules such as hydrogen (Yoshinari and Takano, 2017). Hydrogen is commonly coupled to potassium and sodium transport by different ATPases and other SLCs^5^; hence, this could be a possible explanation to the increased boron in the hemolymph. The link between ion concentration and the cells’ ability to regulate electrochemical balance could also possibly be the explanation to the decrease in cadmium and the increase in cobalt. Generally, an increase in osmotic molecules and disturbed water homeostasis worsen the condition of the flies; water will be transported to the extracellular compartments, the electrolyte balance will be altered, and ion-coupled transporters and pumps would be affected (Castillo et al., 2015). This is observed among the RNA sequencing results, where both sodium/neurotransmitter symporter (Gat, SLC6A1 in humans) and sodium/substrate co-transporter (CG9657) were found to be upregulated.

The phenomena of accumulation of pigments and fluid in the Malpighian tubules have been observed earlier for the knockdown of genes important for transport and trafficking, e.g., *w*, *cho*, *ma*, *mah*, and *red*. Many of them are involved directly in the biosynthesis or the transport of components within the biosynthesis of eye pigments in flies (Lloyd et al., 1998; Grant et al., 2016). For example, knockdown of *Vps16A* (vacuolar protein sorting 16A, also known as *ma*), a gene that is a part of the HOPS complex that together with Syntaxin 17 mediates autophagosome–lysosome fusion (Pulipparacharuvil et al., 2005; Jiang et al., 2014), leads to the accumulation of yellow pigments in Malpighian tubules (Bridges, 1918). Similar pigment accumulation was observed for our knockdown flies, but the fluid accumulation was rather located to the abdominal cavity and not in the Malpighian tubules. Together with the findings that UNC93A affects viral budding due to errors in the endosomal trafficking process (Campbell et al., 2011), it is suggested that *CG4928* could be involved in organelle trafficking. How this is linked to the theory of being a regulator of potassium channels is unclear, but potassium is also of significance for vesicle release, and therefore it is possible that the accumulation is due to dysfunctional vesicle release from, e.g., the autophagosome and/or the lysosome. Further experiments are needed to establish that mechanism. Importantly, the Malpighian tubules are vital for detoxication (Chahine and O’Donnell, 2011), and a recent study presented that *CG4928* is linked to manganese toxicity and might be involved in the process of detoxification (Mohr et al., 2018), and the human UNC93A was recently identified as a metabolite-associated locus in chronic kidney disease patients (Schlosser et al., 2020). Both findings suggest a role in the renal system, and it is clear that *CG4928* affects the function of the renal system in flies.

The orthologs to the human TASK^1^ channels, *Task6* and *Task7*, are lowly expressed in the body, while *CG4928* is expressed throughout the body of both larvae and adult flies. Neither of the two potassium channels was found to have altered mRNA expression as a response to *CG4928* knockdown in RNA sequencing, suggesting that CG4928 does not regulate them by transcriptional regulation. Other potassium channels, e.g., *Irk3*, have a higher specific expression in the Malpighian tubules (Wang et al., 2004) and have been suggested to have a more unique role in the Malpighian tubules compared to *Task6 and Task7*. However, other potassium channels with low expression have been documented in the tubule and been shown to accomplish important functions there (Dow et al., 1994; Wang et al., 2004; Gautam et al., 2017). Furthermore, *Irk3* is an inward-rectifying potassium channel, which means that it passes current into the cell rather than out of the cell, implying that potassium channels such as the TASK^1^ channels could also be needed. It is also possible that CG4928 interacts with different potassium channels, not only the TASK^1^ channels, since the expression distribution of these proteins differs from each other in murine brain tissues (Karschin et al., 2001; Medhurst et al., 2001; Marinc et al., 2014; Ceder et al., 2017).

The disturbances in ion levels and the accumulation of substrates could explain why *CG4928* knockdowns starve faster even if they have high glycogen and glucose levels. Insulin and potassium are tightly connected, where the level of insulin regulates cellular potassium intake, as well as glucose intake (Palmer and Clegg, 2016). Hence, the high levels of glycogen and glucose could be a secondary effect of the altered plasma level of potassium. In addition, dehydration makes the flies more vulnerable to starvation (Albers and Bradley, 2004), and it is possible that the diuretic hormones are altered (Cannell et al., 2016), which are linked to desiccation and starvation resistance. Recently, *CG4928* and UNC93A were shown to be altered by fluctuating glucose levels in flies and primary cortex cell lines from mice (Ceder et al., 2020). In addition, depletion of Kir channels in salivary glands has been shown to alter the performance of the gland and affect sugar feeding in flies (Swale et al., 2017). However, further experiments are needed to reveal the connection between *CG4928* and carbohydrate metabolism and if it is linked to altered potassium levels.

The findings in *D. melanogaster* support the theory that *CG4928* is important for ion and osmotic balance. When reducing *CG4928* expression in flies, it is likely that it disturbs the membrane potential, as observed when overexpressing protein in cells, and/or the ion balance, suggesting that *CG4928* is important for transmembrane ion flows. This finding could also explain why both the *Da-Gal4 > CG4928RNAi GD* line and the *Da-Gal4 > CG4928RNAi KK* line, at temperatures above 29°C, did not produce viable offspring. Both *TASK*^1^ channels and *CG4928* are expressed during the development, and potassium has been found to be of importance for normal development (Haug-Collet et al., 1999; Dahal et al., 2017), indicating that CG4928 most likely is connected to potassium. Furthermore, the salivary gland has an important role for proper pupation and metamorphism (Shirk et al., 1988; Farkas and Mechler, 2000; Takemoto et al., 2007; Wang et al., 2008), and the high expression of CG4928 in that particular tissue could possibly also point out a reason why the offspring died before or during pupation.

In conclusion, CG4928 is an evolutionary conserved protein consisting of 12 transmembrane helices. It co-localizes with TASK^1^ channels, and both overexpression as well as reduction of CG4928 dysregulate cellular processes that involve potassium, e.g., membrane potential in cells and urine production in fruit flies. The findings in this paper suggest that CG4928 could be connected to KCNK3, possibly also KCNK9 and KCNK15. However, structural evidence also points to the possibility that it can perform ion transport over lipid membranes, i.e., being an authentic transporter, where CG4928 expression is linked to proper membrane potential and ion concentrations in the cellular compartments. However, the key mechanisms about the interaction with potassium channels, if CG4928 is an authentic transporter with a substrate that was not identified in this screen or if it has lost its transport function, still remain uncertain. This first in-depth characterization of CG4928 provides a clear example on why orphan transporters are important to study and why gathering of information about them can aid in the understanding about and to establish their role in health and disease.

## Data Availability Statement

The datasets presented in this study can be found in online repositories. The names of the repository/repositories and accession number(s) can be found in the article/Supplementary Material.

## Author Contributions

MC designed the project and experiments, compiled the figures and tables, drafted the manuscript, prepared the material, performed the dissections, RNA and mRNA extraction, cDNA synthesis, qPCR, Ramsay assay, weighing, spectrometry, sodium/potassium measurements, element analysis, starvation assay, locomotory, food intake, and phenotype rescue, captured photos, and modeled the protein structures, RNA sequencing analysis, analysis, cell imaging, analyzing of western blot, analyzed PLA, transport assay, and membrane potential. TA prepared the material, performed dissections, weighing measurements, sugar, tag and protein assays, and membrane potential measurements, assisted in compiling graphs, figures, and Supplementary Figures, and drafted parts of methods. KH performed SH-SY5Y and HEK293T cell cultures and ApoTox^TM^ -GLO measurements, assisted in membrane preparation, performed ICC and PLA, assisted in imaging and compiling of graphs for figures and Supplementary Figures, and drafted parts of methods. VM performed HEK293T cell cultures, membrane preparation, and western blot, and drafted parts of methods. SP prepared material and performed dissections, RNA extractions, cDNA synthesis, and qPCR. EP performed the phylogenetic analysis, compiled the phylogenetic tree and data for Table 1, and drafted parts of methods. MW designed the project and aided in data analysis, experimental setup, and interpretation of results, RF performed RNA sequencing analysis, designed vectors, aided in data analysis, experimental setup, and interpretation of results and drafted the manuscript. All the authors have read and approved the manuscript.

## Conflict of Interest

The authors declare that the research was conducted in the absence of any commercial or financial relationships that could be construed as a potential conflict of interest.

## Footnotes

1 https://www.r-project.org/other-docs.html

2 www.ncbi.nlm.nih.gov/gene

3 www.proteinatlas.org

4 flybase.org

5 slc.bioparadigms.org
