# Supplementary material for: CG4928 Is Vital for Renal Function in Fruit Flies and Membrane Potential in Cells: A First In-Depth Characterization of the Putative Solute Carrier UNC93A
Source: Front Cell Dev Biol. 2020 Oct 14;8:580291. doi: 10.3389/fcell.2020.580291 (PMC7591606; doi:10.3389/fcell.2020.580291)
Supplement: Supplementary file 1 [file Presentation_1.PDF]

Supplementary Material

This file contains Figures S1 to S2; Tables S1 to S3; Legends for Datasets S1 to S4 and SI References.

1 Supplementary Figures and Tables

1.1 Supplementary Figures

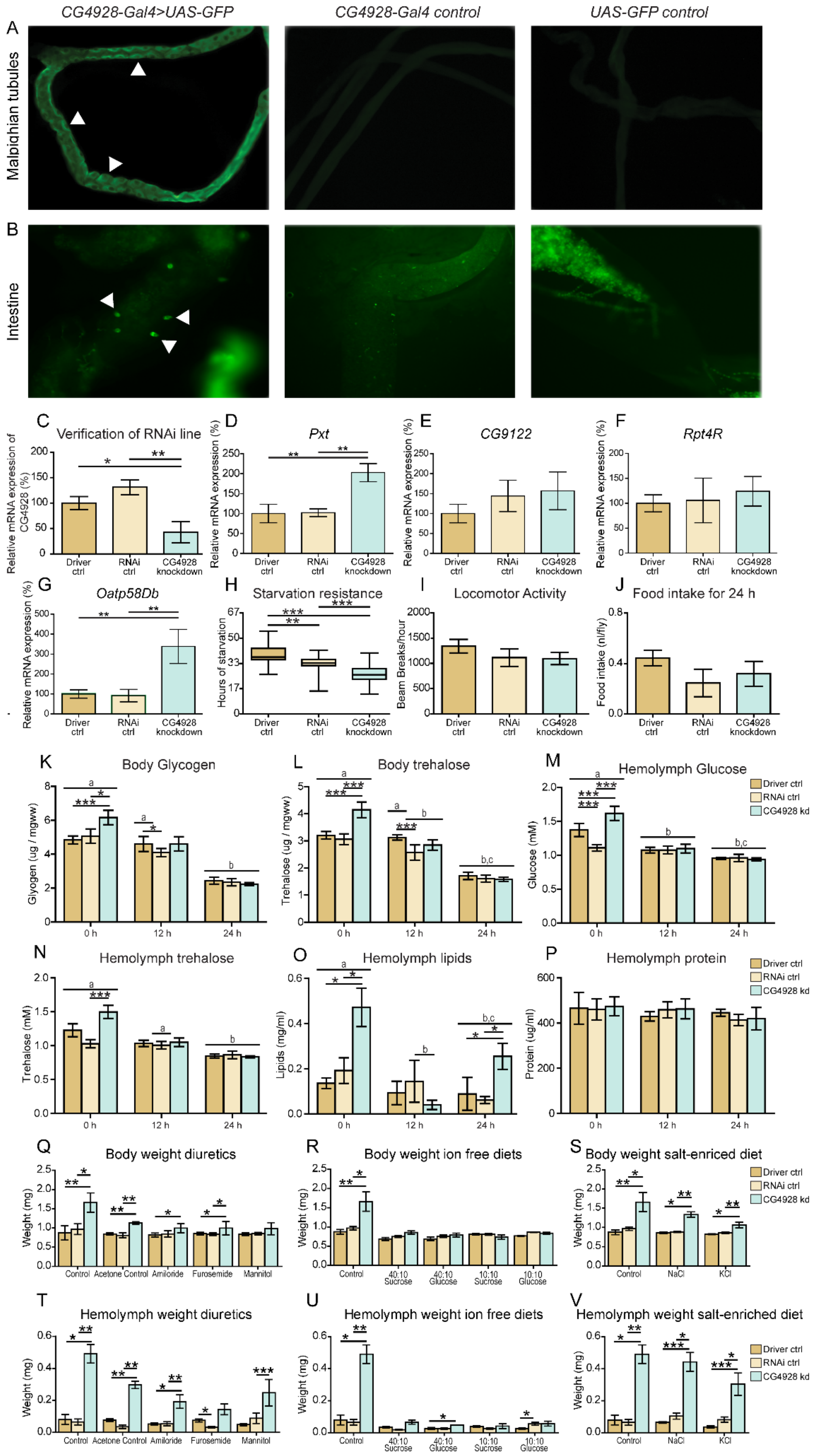
CG4928-Gal4>UAS-GFP
CG4928-Gal4 control
UAS-GFP control
A
Malpighian tubules
B
Intestine
C Verification of RNAi line
D Pxt
E CG9122
F Rpt4R
G Oatp58Db
H Starvation resistance
I Locomotor Activity
J Food intake for 24 h
K Body Glycogen
L Body trehalose
M Hemolymph Glucose
N Hemolymph trehalose
O Hemolymph lipids
P Hemolymph protein
Q Body weight diuretics
R Body weight ion free diets
S Body weight salt-enriced diet
T Hemolymph weight diuretics
U Hemolymph weight ion free diets
V Hemolymph weight salt-enriched diet
Driver ctrl
RNAi ctrl
CG4928 knockdown
CG4928 kd
Relative mRNA expression of CG4928 (%)
Relative mRNA expression (%)
Hours of starvation
Beam Breaks/hour
Food intake (nl/fly)
Glyogen (ug / mgww)
Trehalose (ug / mgww)
Glucose (mM)
Trehalose (mM)
Lipids (mg/ml)
Protein (ug/ml)
Weight (mg)
0 h
12 h
24 h
Control
Acetone Control
Amiloride
Furosemide
Mannitol
40:10 Sucrose
40:10 Glucose
10:10 Sucrose
10:10 Glucose
NaCl
KCl

Supplementary Fig. S1. CG4928 was coupled to green fluorescent protein (GFP) using *CG4928-Gal4* and *Pin/Pin-UAS-GFP*. (A) GFP (CG4928 expression) was found in Malpighian tubules and (B) the intestine of the *CG4928-Gal4>Pin/Pin-UAS-GFP* flies, and not in the two controls. *CG4928* knockdown was verified using qRT-PCR. Normalization was performed against three housekeeping genes (*Actin42a*, *Rpl11* and *Rp49*), relative mRNA expression (±SEM) was plotted, gene expression was compared with both controls; Driver control was set to 100%. Expression differences were calculated using one-way ANOVA and Bonferroni's correction ($*p < 0.05$, $**p < 0.01$, $***p < 0.001$). (C) *CG4928* was knocked down compared with controls. Starvation resistance (n=30) was determined by last beam break per fly and the average resistance in hours was calculated and illustrated in a box plot with upper and lower quartile (Max/Min). Locomotion (n=30) was measured using DAMS, and the average beam break per hour (±SEM) was calculated. Food intake (n=10 for Driver control and RNAi control, n=8 *CG4928* knockdown, with 5 flies in each) was measured with CAFÉ assay, were the average food intake in nl (±SEM) is presented in the graph. Differences were calculated using one-way ANOVA with Bonferroni's corrections ($*p < 0.05$, $**p < 0.01$, $***p < 0.001$). (D) *CG4928* knockdown flies had lower starvation resistance, also RNAi control starve faster compared with Driver control. No differences in (E) activity and (F) food intake were observed. The RNA sequencing was verified by qRT-PCR. Graphs represent mean (±SEM), differences were calculated with Kruskal-Wallis with Mann-Whitney as post-hoc test (*p<0.05, ** p>0.01, ***p>0.001). (G) *Pxt* was upregulated, (H) *CG9122*, the reported off-target gene for *CG4928*, and (I) *Rpt4R* were unaltered and (J) *Oatp58Db* was upregulated. Stored (Body) and circulating (hemolymph) nutrients were quantified in adult males subjected to 0, 12 and 24 hours of starvation (Sugars n=100, TAG and proteins n=30). Graphs represent mean (±SD), difference were analyzed using one-way ANOVA with Bonferroni's corrections (*p<0.05, ** p>0.01, ***p>0.001). In JO, different letters indicate similar groups (i.e. 'a' is different than 'b' and 'b' is different compared with 'c'). At 0 hours of starvation the *CG4928* knockdown had more stored (K) glycogen and (L) trehalose, while no difference was seen at 12 and 24 hours of starvation. *CG4928* knockdown flies had higher levels of (M) circulating glucose at 0 hours of starvation, while no difference was observed at 12 and 24 hours of starvation. Both stored and circulating sugars decreased with the hours of starvation. (N) A difference in trehalose between RNAi control and *CG4928* knockdown at 0 hours was found, while no differences between genotypes were found at 12 or 24 hours of starvation. At 24 hours of starvation the amount of circulating trehalose had decreased compared with 0 hours. (O) *CG4928* knockdowns had higher levels of lipids in their hemolymph at 0 and 24 hours of starvation, and the level of lipids declined with hours of starvation. (P) No difference was observed for proteins in hemolymph. Driver control, RNAi control and *CG4928* knockdown flies were kept on diuretics, sugar:yeast (ion-free) and salt-enriched diets. Body and hemolymph weight were measured (n=30), and the weight was normalized against number of flies. Graphs represent mean (±SD), differences were calculated with Kruskal-Wallis and Dunn's correction (*p<0.05, ** p>0.01, ***p>0.001). (Q) Body weight of flies on standard food, acetone food, Amiloride, Mannitol and Furosemide. (R) All sugar:yeast (ion-free) diets reduced the body weight to the same weight as both controls, while (S) NaCl and KCl enriched diets did not affect the body weight, and *CG4928* knockdown flies were heavier compared with both controls. (T) None of the diuretics reduced the hemolymph weight to the same level as controls. (U) All sugar:yeast diets reduced the hemolymph weight to the same level as both controls, while (V) *CG4928* knockdown flies on salt-enriched diets had higher hemolymph weight compared with controls.

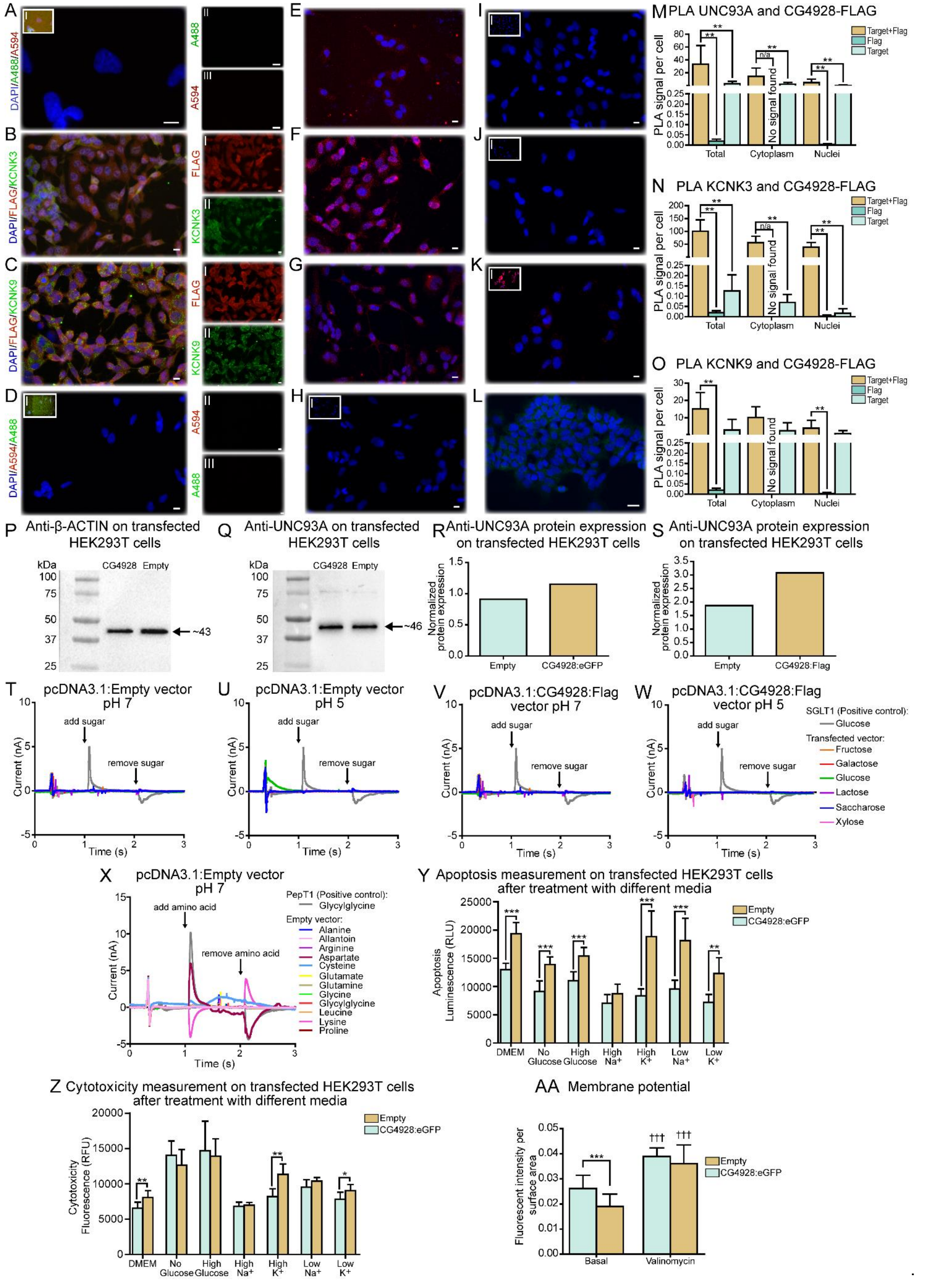

PLA UNC93A and CG4928-FLAG
PLA KCNK3 and CG4928-FLAG
PLA KCNK9 and CG4928-FLAG
Anti-β-ACTIN on transfected HEK293T cells
Anti-UNC93A on transfected HEK293T cells
Anti-UNC93A protein expression on transfected HEK293T cells
pcDNA3.1:Empty vector pH 7
pcDNA3.1:Empty vector pH 5
pcDNA3.1:CG4928:Flag vector pH 7
pcDNA3.1:CG4928:Flag vector pH 5
Apoptosis measurement on transfected HEK293T cells after treatment with different media
Cytotoxicity measurement on transfected HEK293T cells after treatment with different media
Membrane potential

Supplementary Fig. S2. Immunocytochemistry (ICC) for UNC93A, KCNK3 and KCNK9 was performed on wild-type SH-SY5Y cells. (A) A negative control for the secondary antibodies used during ICC was performed and no background signal was observed, (A: I) maximum adjusted brightness. ICC and proximity ligation assay (PLA) were performed on SH-SY5Y cells transfected with 0.5 µg/µl pcDNA3.1-dmCG4928-Flag. Anti-Flag immunostaining in red, KCNK3 and KCNK9 immunostaining in green and nucleus marker in blue (DAPI). Both (B) the anti-FLAG and anti-KCNK3 and (C) anti-Flag and anti-KCNK9 were found to be located in the same cells. (D) Negative control for secondary antibodies used for ICC in (B) and (C), (D:I) maximum adjusted brightness. PLA was performed on transfected SH-SY5Y cells with anti-Flag antibody and anti-UNC93A, anti-KCNK3 or anti-KCNK9. Images were then handled using ImageJ, Fiji edition (73). (E) UNC93A and anti-Flag PLA, (F) anti-KCNK3 and anti-Flag PLA and (G) anti-KCNK9 and anti-Flag PLA, and negative controls for PLA; (H) anti-Flag, (H:I) maximum adjusted brightness, (I) anti-UNC93A, (I:I) maximum adjusted brightness, (J) anti-KCNK3, (J:I) maximum adjusted brightness, and (K) anti-KCNK9, (K:I) maximum adjusted brightness. The PLA signal per cell was calculated for total signals, signals in cytoplasm and nuclei. Duolink^{TM} Image tools (Olink Bioscience) was used to perform the analyzing. GraphPad Prism version 5 was used to retrieve graphs with mean (±SD) and perform statistics. Kruskal-Wallis test with Mann-Whitney as post-hoc test was used to detect differences in PLA signal (*p<0.0491, **p<0.00995, ***p<0.00099). (L) Endogenous UNC93A staining using anti-UNC93A on HEK293T wild type cells. (M) Anti-UNC93A and (N) anti-KCNK3 were co-expressed with anti-Flag, while no co-localization was observed for (O) KCNK9. Transfected HEK293T cells were also used to perform membrane preparation to be used for SSM-based electrophysiology. Western blot was performed on the membrane samples using anti-β-actin and anti-UNC93A. (P) Western Blot for β-actin on CG4928:eGFP vector and empty vector transfected cells, with bands detected at 43 kDa. (Q) Western Blot for anti-UNC93A staining on CG4928:eGFP vector and empty vector transfected cells, with bands detected at 46 kDa. The western blot was quantified using ImageJ, Fiji edition (1), and the protein expression was normalized against β-actin. (R) The CG4928:eGFP and (S) CG4928:Flag vector transfected HEK293T cells were found to have a higher immunostaining of anti-UNC93A after correction against anti-β-actin. SSM-based electrophysiology was performed on cells overexpressing CG4928:eGFP, CG4928:Flag and empty vectors. The membrane was run on three sensors per replicate. Fructose, galactose, glucose, lactose, saccharose or xylose was added to the membranes after 1 second and removed after 2 seconds. SGLT1 was used as a positive control. For amino acids, glycine, glycylglycine, alanine, glutamate, arginine, lysine, leucine, proline, cysteine, glutamine, aspartate and allantoin was added after 1 second and removed after 2 seconds. PepT1 was used as a positive control. The current (nA) was plotted against the time (s) for each compound. No difference was monitored for the empty vector for any of the sugars at neither (S) pH 7 nor (T) pH 5, similar results were obtained for CG4928:Flag transfected cells at (V) pH 7 and (W) pH 5. No difference was observed for (X) empty vector transfected cells and amino acids. The membrane potential was measured using FluoVolt membrane potential kit on CG4928:eGFP and empty vector transfected HEK293T cells. The fluorescent intensity per area were measured for basal membrane potential and after treatment with Valinomycin using ImageJ, Fiji edition (1). (Y) The fluorescent intensity was lower in the CG4928:eGFP transfected cells compared with the control, and after treatment with Valinomycin the fluorescent intensity increased as suspected. Apoptosis and Cytotoxicity were measured on CG4928:eGFP transfected cells and controls (empty vector) after the cells were kept on different meda: DMEM (control media), No glucose DMEM, High glucose DMEM, High NaCl DMEM, High KCl DMEM, Low NaCl media and Low KCl media. (Z) The apoptosis was higher in the control, No glucose, High glucose, High KCl, Low NaCl and Low KCl media, while no difference were observed for High NaCl media. (AA) Cytotoxicity was higher in control, High KCl and Low KCl media, no differences were observed for the other media.

1.2 Supplementary Tables

Supplementary table 1: Ions in the Malpighian tubules were measured using Inductive coupled plasma sector field mass spectrometry. Mean and SD for the triplicates (containing 60 Malpighian tubules in each) for each genotype is presented for each element.

Element	Driver control	RNAi control	CG4928 knockdown
Ag (Silver)	0.007±0.004	0.004±0.003	0.019±0.023
Al (Aluminium)	23.17±7.339	16.65±2.689	19.86±8.194
As (Arsenic)	0.023±0.007	0.036±0.011	0.036±0.014
Au (Gold)	<0.002	<0.002	<0.002
B (Boron)	0.726±0.042	0.633±0.283	5.715±0.733
Ba (Barium)	1.693±0.669	1.515±0.523	0.774±0.373
Be (Beryllium)	<0.005	<0.005	<0.005
Bi (Bismuth)	0.024±0.017	0.047±0.027	0.044±0.034

Br (Bromine)	<2	<2	<2
Ca (Calcium)	1932±2.551	2497±692.0	1600±490.1
Cd (Cadmium)	0.021±0.003	0.032±0.008	0.005±0.003
Ce (Cerium)	0.011±0.015	0.003±0.002	0.002±0.002
Co (Cobalt)	0.145±0.015	0.113±0.022	0.271±0.013
Cr (Chromium)	4.441±7.003	0.362±0.058	0.714±0.741
Ce (Cesium)	0.003±0.002	0.009±0.002	0.006±0.004
Cu (Cupper)	9.590±1.590	6.855±1.888	5.914±1.801
Dy (Dysprosium)	<0.002	<0.002	<0.002
Er (Erbium)	<0.001	<0.001	<0.001
Eu (Europium)	<0.001	<0.001	<0.001
Fe (Iron)	29.22±5.249	35.93±6.960	34.09±14.29
Ga (Gallium)	0.007±0.004	0.006±0.006	0.005±0.003
Gd (Godolonium)	<0.001	<0.001	<0.001
Ge (Germanium)	<0.02	<0.02	<0.02
Hf (Hafnium)	<0.0005	<0.0005	<0.0005
Hg (Mercury)	<0.005	<0.005	<0.005
Ho (Holomium)	<0.0005	<0.0005	<0.0005
I (Iodine)	0.205±0.067	0.324±0.270	0.287±0.145
Ir (Iridium)	<0.0005	<0.0005	<0.0005
K (Potassium)	1389±184.0	1959±200.4	736.6±42.56
La (Lanthanum)	0.007±0.009	0.006±0.002	0.002±0.001
Li (Litium)	12.92±2.426	22.62±4.681	7.850±4.105
Lu (Lutetium)	<0.0005	<0.0005	<0.0005
Mg (Magnesium)	400.8±79.76	467.5±76.34	320.7±32.42
Mn (Manganese)	33.14±9.861	46.01±14.39	33.14±9.861
Mo (Molybdenium)	0.289±0.050	0.390±0.066	0.400±0.194
Na (Sodium)	488.4±130.0	777.0±77.64	898.2±395.5
Nb (Niobium)	<0.001	<0.001	<0.001
Nd (Neodymium)	<0.001	<0.001	<0.001
Ni (Nickel)	0.568±0.067	0.369±0.381	0.450±0.607
Os (Osmium)	<0.0005	<0.0005	<0.0005
P (Phosphourous)	3598±1114	4574±985.6	5745±3729
Pb (Lead)	0.103±0.093	0.203±0.220	0.083±0.075
Pd (Palladium)	<0.001	<0.001	<0.001
Pr (Praseodynium)	<0.001	<0.001	<0.001
Pt (Platinum)	0.080±0.027	0.049±0.029	0.090±0.037
Rb (Rubidium)	1.410±0.400	1.800±0.072	1.295±0.690
Re (Rhenium)	<0.0005	<0.0005	<0.0005
S (Sulfur)	796.1±171.3	1107±195.7	818.1±390.1
Sb (Antimony)	<0.001	<0.001	<0.001
Sc (Scandium)	<0.001	<0.001	<0.001
Se (Selenium)	0.049±0.041	0.089±0.027	0.003±0.001
Si (Silicon)	197.7±57.54	266.1±45.35	210.3±43.50
Sm (Samarium)	<0.001	<0.001	<0.001
Sn (Tin)	0.059±0.021	<0.001	0.041±0.009
Sr (Strontium)	4.359±2.171	4.631±2.781	5.894±5.592
Ta (Tantalum)	<0.001	<0.001	<0.001
Tb (Terebium)	<0.001	<0.001	<0.001
Te (Tellurium)	<0.001	<0.001	<0.001
Th (Thorium)	<0.001	<0.001	<0.001
Ti (Titanium)	0.145±0.028	0.197±0.047	0.784±1.296
Tl (Thallium)	0.004±0.001	0.005±0.001	0.003±0.002
Tm (Thulium)	<0.0005	<0.0005	<0.0005

U (Uranium)	0.019±0.003	0.018±0.001	0.029±0.008
W (Tungsten)	0.283±0.069	0.268±0.140	0.146±0.084
V (Vanadium)	0.008±0.006	0.017±0.019	0.017±0.006
Y (Yttrium)	0.002±0.001	0.002±0.002	0.001±0.001
Yb (Ytterbium)	<0.001	<0.001	<0.001
Zn (Zinc)	26.27±3.734	115.0±24.91	64.71±2.165
Zr (Zirconium)	0.027±0.013	0.017±0.006	0.014±0.007

Supplementary table 2: Transcripts identified in RNA sequencing. Average log2 fold was calculated based on the difference in log2 fold between CG4928 knockdown samples (n=4) and the log2 fold for each control (Driver control, n=4 and RNAi control, n=4). The table summarizes Flybase ID, annotation symbol, name and the average log2 fold.

Flybase ID	Annotation symbol	Dmel/name	Average log2 fold
FBgn0005391	*CG2979*	Yp2	8,19
FBgn0004045	*CG2985*	Yp1	7,64
FBgn0002562	*CG2559*	Lsp1α	4,69
FBgn0085362	*CG34333*	Vml	4,01
FBgn0051089	*CG31089*	CG31089	3,57
FBgn0000427	*CG2175*	dec-1	3,47
FBgn0038469	*CG4009*	CG4009	3,46
FBgn0023495	*CG8823*	Lip3	3,45
FBgn0034151	*CG15617*	CG15617	3,44
FBgn0261987	*CG7660*	Pxt	3,43
FBgn0000357	*CG6517*	Cp18	3,36
FBgn0031141	*CG1304*	CG1304	3,33
FBgn0039685	*CG7592*	Obp99b	3,32
FBgn0004649	*CG1372*	yl	2,80
FBgn0262357	*CG43055*	CG43055	2,71
FBgn0058469	*CR40469*	CR40469	2,65
FBgn0039298	*CG11853*	to	2,64
FBgn0011761	*CG4193*	dhd	2,58
FBgn0087040	*CG8308*	αTub67C	2,54
FBgn0259140	*CG42255*	CG42255	2,51
FBgn0260780	*CG15737*	wisp	2,49
FBgn0000355	*CG6519*	Cp15	2,45
FBgn0085345	*CG34316*	CG34316	2,38
FBgn0034784	*CG9826*	CG9826	2,35
FBgn0032281	*CG17107*	CG17107	2,32
FBgn0038719	*CG16727*	CG16727	2,20
FBgn0037801	*CG3999*	CG3999	2,02
FBgn0039299	*CG11854*	CG11854	2,00
FBgn0039452	*CG14245*	CG14245	2,00
FBgn0039915	*CG1732*	Gat	1,87
FBgn0029950	*CG9657*	CG9657	1,87
FBgn0036880	*CG9295*	Cpr76Bc	1,83
FBgn0036587	*CG4950*	CG4950	1,76
FBgn0038179	*CG9312*	CG9312	1,75
FBgn0053296	*CG33296*	CG33296	1,75
FBgn0027584	*CG4757*	CG4757	1,70
FBgn0250836	*CG8628*	CG8628	1,69
FBgn0030334	*CG4139*	Karl	1,65
FBgn0033720	*CG13160*	CG13160	1,64
FBgn0259896	*CG8942*	NimC1	1,62
FBgn0014033	*CG4099*	Sr-CI	1,62
FBgn0035300	*CG1139*	CG1139	1,62
FBgn0039241	*CG11089*	CG11089	1,61
FBgn0034328	*CG15066*	IM23	1,60
FBgn0035673	*CG6602*	CG6602	1,58
FBgn0037228	*CG1092*	CG1092	1,56

FBgn0040759	*CG13177*	CG13177	1,55
FBgn0052251	*CG32251*	Claspin	1,51
FBgn0035476	*CG12766*	CG12766	1,50
FBgn0037386	*CG1208*	CG1208	1,50
FBgn0037974	*CG12224*	CG12224	1,50
FBgn0000079	*CG18730*	Amy-p	1,49
FBgn0031925	*CG6730*	Cyp4d21	1,46
FBgn0033367	*CG8193*	PPO2	1,46
FBgn0085828	*CR42195*	CR42195	1,45
FBgn0261575	*CG11909*	tobi	1,44
FBgn0029167	*CG7002*	Hml	1,42
FBgn0058198	*CG40198*	CG40198	1,42
FBgn0034329	*CG18108*	IM1	1,39
FBgn0035679	*CG10467*	CG10467	1,36
FBgn0043578	*CG9681*	PGRP-SB1	1,36
FBgn0053012	*CG33012*	CG33012	1,35
FBgn0033296	*CG11669*	Mal-A7	1,34
FBgn0085201	*CG34172*	CG34172	1,34
FBgn0034761	*CG4250*	CG4250	1,32
FBgn0051207	*CG31207*	CG31207	1,31
FBgn0050360	*CG30360*	Mal-A6	1,30
FBgn0037292	*CG2022*	plh	1,30
FBgn0039342	*CG5107*	CG5107	1,29
FBgn0259164	*CG42269*	CG42269	1,28
FBgn0053346	*CG33346*	CG33346	1,27
FBgn0058006	*CG40006*	CG40006	1,25
FBgn0027657	*CG9734*	glob1	1,24
FBgn0051205	*CG31205*	CG31205	1,24
FBgn0051313	*CG31313*	CG31313	1,24
FBgn0033297	*CG8690*	Mal-A8	1,21
FBgn0032283	*CG7296*	CG7296	1,20
FBgn0032253	*CG5322*	LManI	1,18
FBgn0050042	*CG30042*	Cpr49Ab	1,14
FBgn0037714	*CG9396*	CG9396	1,14
FBgn0037697	*CG9363*	GstZ2	1,13
FBgn0011834	*CG2071*	Ser6	1,10
FBgn0267511	*CR45851*	28SrRNA-Ψ:CR45851	1,09
FBgn0037973	*CG18547*	CG18547	1,09
FBgn0002563	*CG4178*	Lsp1β	1,08
FBgn0000078	*CG17876*	Amy-d	1,07
FBgn0004629	*CG8050*	Cys	1,06
FBgn0038115	*CG7966*	CG7966	1,06
FBgn0038412	*CG6898*	Zip89B	1,02
FBgn0034276	*CG6385*	Sardh	1,00
FBgn0029155	*CG5889*	Men-b	0,97
FBgn0038858	*CG5793*	CG5793	0,95
FBgn0041182	*CG7052*	Tep2	0,93
FBgn0053511	*CG33511*	CG33511	0,92
FBgn0085766	*CR40679*	CR40679	0,91
FBgn0029898	*CG14439*	CG14439	0,87
FBgn0036847	*CG11577*	CG11577	0,87
FBgn0262952	*CG34085*	mt:ND4	0,87
FBgn0027843	*CG6906*	CAH2	0,87
FBgn0050489	*CG30489*	Cyp12d1-p	0,83
FBgn0051075	*CG31075*	CG31075	0,79

FBgn0013680	*CG34063*	mt:ND2	0,76
FBgn0259717	*CG42371*	CG42371	0,74
FBgn0037873	*CG6666*	SdhC	0,73
FBgn0031323	*CG5139*	CG5139	-0,72
FBgn0260459	*CG14106*	CG14106	-0,72
FBgn0036031	*CG6761*	CG6761	-0,72
FBgn0032371	*CG4983*	CG4983	-0,77
FBgn0036224	*CG7257*	Rpt4R	-0,81
FBgn0038790	*CG5097*	MtnC	-0,89
FBgn0050094	*CG30094*	CG30094	-0,92
FBgn0052820	*CG32820*	CG32820	-0,92
FBgn0038486	*CG5265*	CG5265	-0,92
FBgn0028870	*CG4691*	CG4691	-0,93
FBgn0261560	*CG8846*	Thor	-0,94
FBgn0037707	*CG16788*	RnpS1	-0,94
FBgn0036704	*CG6497*	CG6497	-0,95
FBgn0001224	*CG4463*	Hsp23	-0,95
FBgn0261445	*CG10072*	sgl	-0,97
FBgn0001230	*CG5436*	Hsp68	-0,99
FBgn0004173	*CG17934*	Mst84Db	-1,00
FBgn0027556	*CG4928*	CG4928	-1,00
FBgn0034846	*CG9863*	CG9863	-1,00
FBgn0010383	*CG6816*	Cyp18a1	-1,01
FBgn0052819	*CG32819*	CG32819	-1,01
FBgn0051901	*CG31901*	Mur29B	-1,02
FBgn0037765	*CG9458*	CG9458	-1,03
FBgn0035004	*CG15874*	Pgam5-2	-1,04
FBgn0036285	*CG10704*	toe	-1,06
FBgn0042127	*CG18789*	CG18789	-1,10
FBgn0263078	*CG43339*	CG43339	-1,10
FBgn0283480	*CG3290*	Alp2	-1,15
FBgn0013725	*CG10108*	phyl	-1,16
FBgn0031034	*CG14205*	CG14205	-1,19
FBgn0086704	*CG31006*	stops	-1,19
FBgn0023214	*CG15085*	edl	-1,21
FBgn0001168	*CG6494*	h	-1,24
FBgn0085489	*CG34460*	CG34460	-1,26
FBgn0032593	*CG5996*	Trpγ	-1,27
FBgn0036767	*CG16775*	CG16775	-1,28
FBgn0039311	*CG10513*	CG10513	-1,29
FBgn0039420	*CG6154*	CG6154	-1,30
FBgn0261814	*CR42756*	CR42756	-1,34
FBgn0038484	*CG5246*	CG5246	-1,36
FBgn0035194	*CG9187*	Psf1	-1,45
FBgn0039091	*CG10182*	CG10182	-1,45
FBgn0036713	*CG6456*	Mip	-1,45
FBgn0028978	*CG5408*	trbl	-1,46
FBgn0265897	*CR44686*	CR44686	-1,52
FBgn0034289	*CG10910*	CG10910	-1,78
FBgn0264515	*CR43914*	CR43914	-1,89
FBgn0036583	*CG13055*	CG13055	-1,95
FBgn0052368	*CG32368*	CG32368	-2,03
FBgn0037204	*CG11131*	CG11131	-2,10
FBgn0266788	*CR45253*	CR45253	-2,23

FBgn0033782	*CG3850*	sug	-2,48
FBgn0034715	*CG3382*	Oatp58Db	-2,51
FBgn0013275	*CG31366*	Hsp70Aa	-2,59
FBgn0266158	*CR44864*	CR44864	-2,80
FBgn0033541	*CG12934*	CG12934	-3,50

Supplementary table 3: Annotation of UNC93A sequences and identified orthologues. All proteomes were obtained from Ensembl, version 86 [1]. Summarize information regarding species, data version, annotated name and sequence accession for each protein sequence identified in the search.

Species	Data version	UNC93A		UNC93B1		MFSD11	
		Name	*Accession*	*Name*	*Accession*	*Name*	*Accession*
A. aegypti	aegypti.AaegL3.pep.all	aaUNC93A.1	AAEL004114-PA.2			aaMFSD11	AAEL013304-PA.1
		aaUNC93A2	AAEL004048-PA.1				
A. carolinensis	AnoCar2.0.pep.all	acUNC93A	ENSACAP00000010177	acUNC93B1	ENSACAP00000008695		
C. elegans	WBcel235.pep.all	unc-93	C46F11.1a			ceMFSD11a	M153.2
						ceMFSD11b	F36G9.3a
						ceMFSD11c	F31D5.1
						ceMFSD11d	Y52E8A.4
						ceMFSD11g	C27C12.4
C. intestinalis	KH.pep.all	ciUnc93a	ENSCINP00000012908			ciMFSD11	ENSCINP00000013214
D. rerio	GRCz10.pep.all	drUNC93A	ENSDARP00000121615	drUNC93B1	ENSDARP00000139372	drMFSD11	ENSDARP00000136716
D. melanogaster	BDGP6.pep.all	CG4928	FBpp0074118			CG18549	FBpp0082101
		CG2121	FBpp0288563				
G. gallus	Galgal4.pep.all	ggUNC93A	ENSGALP00000018482	ggUNC93B1	ENSGALP00000005384	ggMFSD11	ENSGALP00000002730
G. aculeatus	BROADS1.pep.all	gaUNC93A	ENSGACP00000008899	gaUNC93B1	ENSGACP00000024392	gaMFSD11	ENSGACP00000019646
M. musculus	GRCm38.pep.all	mmUNC93A	ENSMUSP00000082032	mmUNC93B1	ENSMUSP00000124272	mmMFSD11	ENSMUSP00000101971

2 Legends for datasets S1 to S4

Dataset S1 (separate file). A total of 232 620 764 transcripts mapped uniquely and was used in subsequent RNA sequencing analysis. The assembled transcripts were used in CuffLinks and Cuffmerge to obtain a final transcriptome assembly. Subsequently Cuffquant and Cuffdiff were used to calculate differential expression and finally the packaged CummRBound were used in R (https://www.r-project.org/other-docs.html) to plot the results. The version for all CuffLinks (11-13) tools were 2.2.1.

Dataset S2 (separate file). Protein sequences for sup-9 from *C. elegans*, KCNK family members from human and orthologues in *D. melanogaster*. All protein sequences were obtained from uniport.org [2].

Dataset S3 (separate file). The epitope of the anti-UNC93A antibody (ab69443, Abcam) binds to an 18 amino acid synthetic peptide from near the carboxy terminus of human Unc93a. Hence a comparison between the CG4928 protein sequences and the human UNC93A, focusing on the carboxy terminus, was performed using the global and local alignment tools EMBOSS NEEDLE and WATER [3].

Dataset S4 (separate file). Sequences inserted when designing the pcDNA3.1-CG4928-Flag (Invitrogen, 17AD6RSP) and pcDNA3.1-CG4928-eGFP (Invitrogen, 17ADONXP) vectors.

3 References

1. Cunningham, F., et al., *Ensembl 2015.* Nucleic Acids Res, 2015. **43**(Database issue): p. D662-9.
2. UniProt, C., *UniProt: a worldwide hub of protein knowledge.* Nucleic Acids Res, 2019. **47**(D1): p. D506-D515.
3. Li, W., et al., *The EMBL-EBI bioinformatics web and programmatic tools framework.* Nucleic Acids Res, 2015. **43**(W1): p. W580-4.
