# Supplementary material for: CG4928 Is Vital for Renal Function in Fruit Flies and Membrane Potential in Cells: A First In-Depth Characterization of the Putative Solute Carrier UNC93A
Source: Front Cell Dev Biol. 2020 Oct 14;8:580291. doi: 10.3389/fcell.2020.580291 (PMC7591606; doi:10.3389/fcell.2020.580291)
Supplement: Supplementary file 4 [file Data_Sheet_3.PDF]

Supplementary data: Sequences inserted in the pcDNA3.1 vector

pcDNA3.1-CG4928-Flag (Invitrogen, 17AD6RSP)

GAATTCATGGACTACAAAGACGATGACGACAAGACTGGTTTCACGAACGCCGGTTTCGAGAACGATG
AGCCCGTCAAGCCAAAAGCTGGTTTCGAGCCCGATACCGCCTCGCTGCGAGAGAAAGTTGTGCTGAAT
CCGGGCGAGAAATGGCGCATCTTAAAGAATATCTCGATCATCTCGATCGCCTTCATGGTGCAGTTTAC
AGCGTTTCAGGGCACGGCCAATCTGCAATCCTCTATAAACGCAAAGGATGGCCTGGGCACAGTGTCGC
TTAGTGCGATCTACGCCGCCTTGGTGGTCTCCTGCATCTTCCTGCCCACTCTGATCATTAGGAAACTAA
CGGTCAAATGGACATTGGTGTGCAGCATGCTCTGCTACGCACCATACATCGCATTCCAGCTCTTCCCGC
GATTCTACACGTTGGTGCCCGCCGGTATCCTTGTGGGTATGGGCGCCGCACCCATGTGGGCGTCCAAG
GCCACCTATTTGACGCAGGTTGGACAGGTGTATGCCAAGATCACGGAACAGGCGGTGGATGCCATTAT
AGTGCGATTCTTTGGCTTCTTCTTCCTGGCCTGGCAATCCGCTGAGCTCTGGGGCAATCTCATCTCCAG
TTTGGTCCTCTCCAGCGGCGCTCATGGCGGTGGCAGCAGCTCGAACACGACGGTCAGCGAGGAGGATT
TGCAGTTCTGCGGTGCCAACTTCTGCACCACCGGAAGCGGCGGCCATGGCAACCTGGAGCGTCCGCCA
GAGGATGAGATCTTCGAGATCTCGATGATCTATCTGTCCTGCATTGTGGCCGCCGTCTGCATCATCGCC
TTCTTCTTGGATCCCCTCAAGCGGTATGGTGAGAAGCGCAAGGGCTCCAATTCTGCCGCCGAGTTGTCC
GGACTGCAGCTGCTGTCCGCCACCTTCCGCCAGATGAAGAAACCGAATCTGCAGCTCCTCATCCCTAT
CACCGTTTTCATTGGCATGGAGCAGGCTTTCATCGGTGCCGATTTCACTCAGGCCTATGTGGCCTGCGC
CCTGGGAGTGAACAAGATTGGCTTCGTCATGATCTGTTTCGGTGTGGTGAATGCCCTCTGCTCGATCCT
CTTCGGATCGGTAATGAAGTATATCGGCCGCACGCCCATCATTGTACTGGGCGCCGTCGTCCACTTCAC
CCTGATCACCGTTGAGCTCTTCTGGCGCCCCAATCCCGATAATCCCATCATCTTCTATGCCATGTCCGG
CCTGTGGGGCGTCGGCGATGCCGTGTGGCAGACCCAGATCAACGGACTGTACGGACTGCTGTTCCGCA
GGAACAAGGAGGCTGCCTTCTCCAACTACCGCCTATGGGAGTCCGCCGGATTCGTTATTGCCTACGCC
TATGCCACAACACTCTGCACGCAGATGAAGCTCTACATTCTGCTGGCTGTACTCACGCTCGGCTGCATC
GGCTACGTGATTGTGGAGATCCTGTACAGGAAGAAGCAACGCAAGCTCAAGAAGCAGGAGAAGCTGG
AGGCCGCGGAGAAGGAGAAGGAGGCCGCCGCCGCCGCAGCCGCCGCCGCTTTGGCCGCCGCCGAGGC
AGGAGCAGATGGCGTCGAGGAGACCGACGACGAGCTGGACGATCTCGAGGAGGACATTGTGGTCACG
CGCCTGCATCATCATCATCATCATCATTAACTCGAG

pcDNA3.1-CG4928-eGFP (Invitrogen,17ADONXP)

ATGACTGGTTTCACGAACGCCGGTTTCGAGAACGATGAGCCCGTCAAGCCAAAAGCTGGTTTCGAGCC
CGATACCGCCTCGCTGCGAGAGAAAGTTGTGCTGAATCCGGGCGAGAAATGGCGCATCTTAAAGAAT
ATCTCGATCATCTCGATCGCCTTCATGGTGCAGTTTACAGCGTTTCAGGGCACGGCCAATCTGCAATCC
TCTATAAACGCAAAGGATGGCCTGGGCACAGTGTCGCTTAGTGCGATCTACGCCGCCTTGGTGGTCTC
CTGCATCTTCCTGCCCACTCTGATCATTAGGAAACTAACGGTCAAATGGACATTGGTGTGCAGCATGCT
CTGCTACGCACCATACATCGCATTCCAGCTCTTCCCGCGATTCTACACGTTGGTGCCCGCCGGTATCCT
TGTGGGTATGGGCGCCGCACCCATGTGGGCGTCCAAGGCCACCTATTTGACGCAGGTTGGACAGGTGT
ATGCCAAGATCACGGAACAGGCGGTGGATGCCATTATAGTGCGATTCTTTGGCTTCTTCTTCCTGGCCT
GGCAATCCGCTGAGCTCTGGGGCAATCTCATCTCCAGTTTGGTCCTCTCCAGCGGCGCTCATGGCGGTG
GCAGCAGCTCGAACACGACGGTCAGCGAGGAGGATTTGCAGTTCTGCGGTGCCAACTTCTGCACCACC
GGAAGCGGCGGCCATGGCAACCTGGAGCGTCCGCCAGAGGATGAGATCTTCGAGATCTCGATGATCT
ATCTGTCCTGCATTGTGGCCGCCGTCTGCATCATCGCCTTCTTCTTGGATCCCCTCAAGCGGTATGGTG
AGAAGCGCAAGGGCTCCAATTCTGCCGCCGAGTTGTCCGGACTGCAGCTGCTGTCCGCCACCTTCCGC
CAGATGAAGAAACCGAATCTGCAGCTCCTCATCCCTATCACCGTTTTCATTGGCATGGAGCAGGCTTTC
ATCGGTGCCGATTTCACTCAGGCCTATGTGGCCTGCGCCCTGGGAGTGAACAAGATTGGCTTCGTCAT
GATCTGTTTCGGTGTGGTGAATGCCCTCTGCTCGATCCTCTTCGGATCGGTAATGAAGTATATCGGCCG
CACGCCCATCATTGTACTGGGCGCCGTCGTCCACTTCACCCTGATCACCGTTGAGCTCTTCTGGCGCCC
CAATCCCGATAATCCCATCATCTTCTATGCCATGTCCGGCCTGTGGGGCGTCGGCGATGCCGTGTGGCA
GACCCAGATCAACGGACTGTACGGACTGCTGTTCCGCAGGAACAAGGAGGCTGCCTTCTCCAACTACC
GCCTATGGGAGTCCGCCGGATTCGTTATTGCCTACGCCTATGCCACAACACTCTGCACGCAGATGAAG
CTCTACATTCTGCTGGCTGTACTCACGCTCGGCTGCATCGGCTACGTGATTGTGGAGATCCTGTACAGG

AAGAAGCAACGCAAGCTCAAGAAGCAGGAGAAGCTGGAGGCCGCGGAGAAGGAGAAGGAGGCCGCC
GCCGCCGCAGCCGCCGCCGCTTTGGCCGCCGCCGAGGCAGGAGCAGATGGCGTCGAGGAGACCGACG
ACGAGCTGGACGATCTCGAGGAGGACATTGTGGTCACGCGCCTGTAACCCCCCCCCCTAACGTTACTG
GCCGAAGCCGCTTGGAATAAGGCCGGTGTGCGTTTGTCTATATGTTATTTTCCACCATATTGCCGTCTT
TTGGCAATGTGAGGGCCCGGAAACCTGGCCCTGTCTTCTTGACGAGCATTCCTAGGGGTCTTTCCCCTC
TCGCCAAAGGAATGCAAGGTCTGTTGAATGTCGTGAAGGAAGCAGTTCCTCTGGAAGCTTCTTGAAGA
CAAACAACGTCTGTAGCGACCCTTTGCAGGCAGCGGAACCCCCCACCTGGCGACAGGTGCCTCTGCGG
CCAAAAGCCACGTGTATAAGATACACCTGCAAAGGCGGCACAACCCCAGTGCCACGTTGTGAGTTGG
ATAGTTGTGGAAAGAGTCAAATGGCTCTCCTCAAGCGTATTCAACAAGGGGCTGAAGGATGCCCAGA
AGGTACCCCATTGTATGGGATCTGATCTGGGGCCTCGGTGCACATGCTTTACATGTGTTTAGTCGAGGT
TAAAAAACGTCTAGGCCCCCCGAACCACGGGGACGTGGTTTTCCTTTGAAAAACACGATGATAATATG
GTGAGCAAGGGCGAGGAGCTGTTCACCGGGGTGGTGCCCATCCTGGTCGAGCTGGACGGCGACGTAA
ACGGCCACAAGTTCAGCGTGTCCGGCGAGGGCGAGGGCGATGCCACCTACGGCAAGCTGACCCTGAA
GTTCATCTGCACCACCGGCAAGCTGCCCGTGCCCTGGCCCACCCTCGTGACCACCCTGACCTACGGCGT
GCAGTGCTTCAGCCGCTACCCCGACCACATGAAGCAGCACGACTTCTTCAAGTCCGCCATGCCCGAAG
GCTACGTCCAGGAGCGCACCATCTTCTTCAAGGACGACGGCAACTACAAGACCCGCGCCGAGGTGAA
GTTCGAGGGCGACACCCTGGTGAACCGCATCGAGCTGAAGGGCATCGACTTCAAGGAGGACGGCAAC
ATCCTGGGGCACAAGCTGGAGTACAACTACAACAGCCACAACGTCTATATCATGGCCGACAAGCAGA
AGAACGGCATCAAGGTGAACTTCAAGATCCGCCACAACATCGAGGACGGCAGCGTGCAGCTCGCCGA
CCACTACCAGCAGAACACCCCCATCGGCGACGGCCCCGTGCTGCTGCCCGACAACCACTACCTGAGCA
CCCAGTCCGCCCTGAGCAAAGACCCCAACGAGAAGCGCGATCACATGGTCCTGCTGGAGTTCGTGACC
GCCGCCGGGATCACTCTCGGCATGGACGAGCTGTACAAG
