# Supplementary material for: CG4928 Is Vital for Renal Function in Fruit Flies and Membrane Potential in Cells: A First In-Depth Characterization of the Putative Solute Carrier UNC93A
Source: Front Cell Dev Biol. 2020 Oct 14;8:580291. doi: 10.3389/fcell.2020.580291 (PMC7591606; doi:10.3389/fcell.2020.580291)
Supplement: Supplementary file 5 [file Data_Sheet_4.PDF]

Supplementary data: Epitope comparison

Sequences

- CG4928-PA (Flybase.org ID FBpp0074118)

MTGFTNAGFENDEPVKPKAGFEPDTASLREKVVLNPGEKWRILKNISIISIAFMVQFTAFQGTANLQS
SINAKDGLGTVSLSAIYAALVVSCIFLPTLIIRKLTVKWTLVCSMLCYAPYIAFQLFPRFYTLVPAGI
LVGMGAAPMWASKATYLTQVGQVYAKITEQAVDAIIVRFFGFFFLAWQSAELWGNLISSLVLSSGAHG
GGSSSNTTVSEEDLQFCGANFCTTGSGGHGNLERPPEDEIFEISMIYLSCIVAAVCIIAFFLDPLKRY
GEKRKGSNSAAELSGLQLLSATFRQMKKPNLQLLIPITVFIGMEQAFIGADFTQAYVACALGVNKIGF
VMICFGVVNALCSILFGSVMKYIGRTPIIVLGAVVHFTLITVELFWRPNPDNPIIFYAMSGLWGVGDA
VWQTQINGLYGLLFRRNKEAAFSNYRLWESAGFVIAYAYATTLCTQMKLYILLAVLTLGCIGYVIVEI
LYRKKQRKLKKQEKLEAAEKEKEAAAAAAAAALAAAEAGADGVEETDDELDDLEEDIVVTRL

- CG4928-PB (Flybase.org ID FBpp0074119)

MTGFTNAGFENDEPVKPKAGFEPDTASLREKVVLNPGEKWRILKNISIISIAFMVQFTAFQGTANLQS
SINAKDGLGTVSLSAIYAALVVSCIFLPTLIIRKLTVKWTLVCSMLCYAPYIAFQLFPRFYTLVPAGI
LVGMGAAPMWASKATYLTQVGQVYAKITEQAVDAIIVRFFGFFFLAWQSAELWGNLISSLVLSSGAHG
GGSSSNTTVSEEDLQFCGANFCTTGSGGHGNLERPPEDEIFEISMIYLSCIVAAVCIIAFFLDPLKRY
GEKRKGSNSAAELSGLQLLSATFRQMKKPNLQLLIPITVFIGMEQAFIGADFTQAYVACALGVNKIGF
VMICFGVVNALCSILFGSVMKYIGRTPIIVLGAVVHFTLITVELFWRPNPDNPIIFYAMSGLWGVGDA
VWQTQINGLYGLLFRRNKEAAFSNYRLWESAGFVIAYAYATTLCTQMKLYILLAVLTLGCIGYVIVEI
LYRKKQRKLKKQEKLEAAEKEKEAAAAAAAAALAAAEAGADGVEETDDELDDLEEDIVVTRL

- CG4928-PC (Flybase.org ID FBpp0309738)

MTGFTNAGFENDEPVKPKAGFEPDTASLREKVVLNPGEKWRILKNISIISIAFMVQFTAFQGTANLQS
SINAKDGLGTVSLSAIYAALVVSCIFLPTLIIRKLTVKWTLVCSMLCYAPYIAFQLFPRFYTLVPAGI
LVGMGAAPMWASKATYLTQVGQVYAKITEQAVDAIIVRFFGFFFLAWQSAELWGNLISSLVLSSGAHG
GGSSSNTTVSEEDLQFCGANFCTTGSGGHGNLERPPEDEIFEISMIYLSCIVAAVCIIAFFLDPLKRY
GEKRKGSNSAAELSGLQLLSATFRQMKKPNLQLLIPITVFIGMEQAFIGADFTQAYVACALGVNKIGF
VMICFGVVNALCSILFGSVMKYIGRTPIIVLGAVVHFTLITVELFWRPNPDNPIIFYAMSGLWGVGDA
VWQTQINGLYGLLFRRNKEAAFSNYRLWESAGFVIAYAYATTLCTQMKLYILLAVLTLGCIGYVIVEI
LYRKKYLISRKYKISFFLS

- Human UNC93A (Uniprot.org ID Q86WB7)

MDRSLRNVLVVSFGFLLLFTAYGGLQSLQSSLYSEEGLGVTALSTLYGGMLLSSMFLPPLLIERLGCK
GTIILSMCGYVAFSVGNFFASWYTLIPTSILLGLGAAPLWSAQCTYLTITGNTHAEKAGKRGKDMVNQ
YFGIFFLIFQSSGVWGNLISSLVFGQTPSQETLPEEQLTSCGASDCLMATTTTNSTQRPSQQLVYTLL
GIYTGSGVLAVLMIAAFLQPIRDVQRESEGEKKSVPFWSTLLSTFKLYRDKRLCLLILLPLYSGLQQG
FLSSEYTRSYVTCTLGIQFVGYVMICFSATDALCSVLYGKVSQYTGRAVLYVLGAVTHVSCMIALLLW
RPRADHLAVFFVFSGLWGVADAVWQTQNNALYGVLFEKSKEAAFANYRLWEALGFVIAFGYSMFLCVH
VKLYILLGVLSLTMVAYGLVECVESKNPIRPHAPGQVNQAEDEEIQTKM

Pair-wise global alignment

- Human UNC93A and CG4928-PA

Identity: 187/538 (34.8 %)
Similarity: 287/538 (53.3 %)
Gaps: 81/538 (15.1 %)

```
HUMAN                  1 --------------------------------------MDRSLRNVLVVS     12
                                                               ..|.|:|:.::|
CG4928-PA              1 MTGFTNAGFENDEPVKPKAGFEPDTASLREKVVLNPGEKWRILKNISIIS     50
```

```
HUMAN              13 FGFLLLFTAYGGLQSLQSSLYSEEGLGVTALSTLYGGMLLSSMFLPPLLI      62
                      ..|::.|||:.|..:||||:.:::|||..:||.:|..:::|.:|||.|:|
CG4928-PA          51 IAFMVQFTAFQGTANLQSSINAKDGLGTVSLSAIYAALVVSCIFLPTLII     100

HUMAN              63 ERLGCKGTIILSMCGYVAFSVGNFFASWYTLIPTSILLGLGAAPLWSAQC     112
                      .:|..|.|::.||..|..:.....|..:|||:|..||:|:||||:|:::.
CG4928-PA         101 RKLTVKWTLVCSMLCYAPYIAFQLFPRFYTLVPAGILVGMGAAPMWASKA     150

HUMAN             113 TYLTITGNTHAEKAGKRGKDMVNQYFGIFFLIFQSSGVWGNLISSLVF--     160
                      ||||..|..:|:...:....::.::||.|||.:||:.:|||||||||.
CG4928-PA         151 TYLTQVGQVYAKITEQAVDAIIVRFFGFFFLAWQSAELWGNLISSLVLSS     200

HUMAN             161 ----GQTPSQETLPEEQLTSCGASDCLMATTTTNSTQRPSQQLVYTLLGI     206
                          |.:.|..|:.||.|..|||:.|...:....:.:||.:..::.:..|
CG4928-PA         201 GAHGGGSSSNTTVSEEDLQFCGANFCTTGSGGHGNLERPPEDEIFEISMI     250

HUMAN             207 YTGSGVLAVLMIAAFLQPIRDVQRESEGEKKSVPF--WSTLLSTFKLYRD     254
                      |....|.||.:||.||.|::....:.:|...:...  ...|.:||:..:.
CG4928-PA         251 YLSCIVAAVCIIAFFLDPLKRYGEKRKGSNSAAELSGLQLLSATFRQMKK     300

HUMAN             255 KRLCLLILLPLYSGLQQGFLSSEYTRSYVTCTLGIQFVGYVMICFSATDA     304
                      ..|.|||.:.::.|::|.|:.:::|::||.|.||:..:|:|||||...:|
CG4928-PA         301 PNLQLLIPITVFIGMEQAFIGADFTQAYVACALGVNKIGFVMICFGVVNA     350

HUMAN             305 LCSVLYGKVSQYTGRAVLYVLGAVTHVSCMIALLLWRPRADHLAVFFVFS     354
                      |||:|:|.|.:|.||..:.|||||.|.:.:...|.|||..|:..:|:..|
CG4928-PA         351 LCSILFGSVMKYIGRTPIIVLGAVVHFTLITVELFWRPNPDNPIIFYAMS     400

HUMAN             355 GLWGVADAVWQTQNNALYGVLFEKSKEAAFANYRLWEALGFVIAFGYSMF     404
                      |||||.|||||||.|.|||:||.::|||||:||||||:.|||||:.|:..
CG4928-PA         401 GLWGVGDAVWQTQINGLYGLLFRRNKEAAFSNYRLWESAGFVIAYAYATT     450

HUMAN             405 LCVHVKLYILLGVLSLTMVAYGLVECVESKNPIRPHAPGQVNQAEDEEIQ     454
                      ||..:||||||.||:|..:.|.:||.:..|...:.....::..||.|:..
CG4928-PA         451 LCTQMKLYILLAVLTLGCIGYVIVEILYRKKQRKLKKQEKLEAAEKEKEA     500

HUMAN             455 TKM-----------------------------------    457
                      ...
CG4928-PA         501 AAAAAAAALAAAEAGADGVEETDDELDDLEEDIVVTRL    538
```

- Human UNC93A and CG4928-PB

Identity: 187/538 (34.8 %)
Similarity: 287/538 (53.3 %)
Gaps: 81/538 (15.1 %)

```
HUMAN               1 ---------------------------------------MDRSLRNVLVVS      12
                                                            ..|.|:|:.::|
CG4928-PB           1 MTGFTNAGFENDEPVKPKAGFEPDTASLREKVVLNPGEKWRILKNISIIS      50

HUMAN              13 FGFLLLFTAYGGLQSLQSSLYSEEGLGVTALSTLYGGMLLSSMFLPPLLI      62
                      ..|::.|||:.|..:||||:.:::|||..:||.:|..:::|.:|||.|:|
CG4928-PB          51 IAFMVQFTAFQGTANLQSSINAKDGLGTVSLSAIYAALVVSCIFLPTLII     100

HUMAN              63 ERLGCKGTIILSMCGYVAFSVGNFFASWYTLIPTSILLGLGAAPLWSAQC     112
                      .:|..|.|::.||..|..:.....|..:|||:|..||:|:||||:|:::.
CG4928-PB         101 RKLTVKWTLVCSMLCYAPYIAFQLFPRFYTLVPAGILVGMGAAPMWASKA     150

HUMAN             113 TYLTITGNTHAEKAGKRGKDMVNQYFGIFFLIFQSSGVWGNLISSLVF--     160
                      ||||..|..:|:...:....::.::||.|||.:||:.:|||||||||.
CG4928-PB         151 TYLTQVGQVYAKITEQAVDAIIVRFFGFFFLAWQSAELWGNLISSLVLSS     200

HUMAN             161 ----GQTPSQETLPEEQLTSCGASDCLMATTTTNSTQRPSQQLVYTLLGI     206
                          |.:.|..|:.||.|..|||:.|...:....:.:||.:..::.:..|
CG4928-PB         201 GAHGGGSSSNTTVSEEDLQFCGANFCTTGSGGHGNLERPPEDEIFEISMI     250

HUMAN             207 YTGSGVLAVLMIAAFLQPIRDVQRESEGEKKSVPF--WSTLLSTFKLYRD     254
```

```
                     |....|.||.:||.||.|::....:.:|...:...  ...|.:||:..:.
CG4928-PB        251 YLSCIVAAVCIIAFFLDPLKRYGEKRKGSNSAAELSGLQLLSATFRQMKK    300

HUMAN            255 KRLCLLILLPLYSGLQQGFLSSEYTRSYVTCTLGIQFVGYVMICFSATDA    304
                     ..|.|||.:.::.|::|.|:.:::|::||.|.||:..:|:|||||...:|
CG4928-PB        301 PNLQLLIPITVFIGMEQAFIGADFTQAYVACALGVNKIGFVMICFGVVNA    350

HUMAN            305 LCSVLYGKVSQYTGRAVLYVLGAVTHVSCMIALLLWRPRADHLAVFFVFS    354
                     |||:|:|.|.:|.||..:.|||||.|.:.:...|.|||..|:..:|:..|
CG4928-PB        351 LCSILFGSVMKYIGRTPIIVLGAVVHFTLITVELFWRPNPDNPIIFYAMS    400

HUMAN            355 GLWGVADAVWQTQNNALYGVLFEKSKEAAFANYRLWEALGFVIAFGYSMF    404
                     |||||.|||||||.|.|||:||.::|||||:||||||:.|||||:.|:..
CG4928-PB        401 GLWGVGDAVWQTQINGLYGLLFRRNKEAAFSNYRLWESAGFVIAYAYATT    450

HUMAN            405 LCVHVKLYILLGVLSLTMVAYGLVECVESKNPIRPHAPGQVNQAEDEEIQ    454
                     ||..:||||||.||:|..:.|.:||.:..|...:.....::..||.|:..
CG4928-PB        451 LCTQMKLYILLAVLTLGCIGYVIVEILYRKKQRKLKKQEKLEAAEKEKEA    500

HUMAN            455 TKM-----------------------------------    457
                     ...
CG4928-PB        501 AAAAAAAALAAAEAGADGVEETDDELDDLEEDIVVTRL    538
```

- Human UNC93A and CG4928-PC

Identity: 186/510 (36.5 %)
Similarity: 283/510 (55.5 %)
Gaps: 68/510 (13.3 %)

```
HUMAN              1 --------------------------------------MDRSLRNVLVVS     12
                                                           ..|.|:|:.::|
CG4928-PC          1 MTGFTNAGFENDEPVKPKAGFEPDTASLREKVVLNPGEKWRILKNISIIS     50

HUMAN             13 FGFLLLFTAYGGLQSLQSSLYSEEGLGVTALSTLYGGMLLSSMFLPPLLI     62
                     ..|::.|||:.|..:||||:.:::|||..:||.:|..:::|.:|||.|:|
CG4928-PC         51 IAFMVQFTAFQGTANLQSSINAKDGLGTVSLSAIYAALVVSCIFLPTLII    100

HUMAN             63 ERLGCKGTIILSMCGYVAFSVGNFFASWYTLIPTSILLGLGAAPLWSAQC    112
                     .:|..|.|::.||..|..:.....|..:|||:|..||:|:||||:|:::.
CG4928-PC        101 RKLTVKWTLVCSMLCYAPYIAFQLFPRFYTLVPAGILVGMGAAPMWASKA    150

HUMAN            113 TYLTITGNTHAEKAGKRGKDMVNQYFGIFFLIFQSSGVWGNLISSLVF--    160
                     ||||..|..:|:...:....::.::||.|||.:||:.:|||||||||.
CG4928-PC        151 TYLTQVGQVYAKITEQAVDAIIVRFFGFFFLAWQSAELWGNLISSLVLSS    200

HUMAN            161 ----GQTPSQETLPEEQLTSCGASDCLMATTTTNSTQRPSQQLVYTLLGI    206
                         |.:.|..|:.||.|..|||:.|...:....:.:||.:..::.:..|
CG4928-PC        201 GAHGGGSSSNTTVSEEDLQFCGANFCTTGSGGHGNLERPPEDEIFEISMI    250

HUMAN            207 YTGSGVLAVLMIAAFLQPIRDVQRESEGEKKSVPF--WSTLLSTFKLYRD    254
                     |....|.||.:||.||.|::....:.:|...:...  ...|.:||:..:.
CG4928-PC        251 YLSCIVAAVCIIAFFLDPLKRYGEKRKGSNSAAELSGLQLLSATFRQMKK    300

HUMAN            255 KRLCLLILLPLYSGLQQGFLSSEYTRSYVTCTLGIQFVGYVMICFSATDA    304
                     ..|.|||.:.::.|::|.|:.:::|::||.|.||:..:|:|||||...:|
CG4928-PC        301 PNLQLLIPITVFIGMEQAFIGADFTQAYVACALGVNKIGFVMICFGVVNA    350

HUMAN            305 LCSVLYGKVSQYTGRAVLYVLGAVTHVSCMIALLLWRPRADHLAVFFVFS    354
                     |||:|:|.|.:|.||..:.|||||.|.:.:...|.|||..|:..:|:..|
CG4928-PC        351 LCSILFGSVMKYIGRTPIIVLGAVVHFTLITVELFWRPNPDNPIIFYAMS    400

HUMAN            355 GLWGVADAVWQTQNNALYGVLFEKSKEAAFANYRLWEALGFVIAFGYSMF    404
                     |||||.|||||||.|.|||:||.::|||||:||||||:.|||||:.|:..
CG4928-PC        401 GLWGVGDAVWQTQINGLYGLLFRRNKEAAFSNYRLWESAGFVIAYAYATT    450

HUMAN            405 LCVHVKLYILLGVLSLTMVAYGLVECVESKNPIRPHAPGQVNQAEDEEIQ    454
                     ||..:||||||.||:|..:.|.:||.:..|..:               |.
```

```
CG4928-PC        451 LCTQMKLYILLAVLTLGCIGYVIVEILYRKKYL---------------IS    485

HUMAN            455 TKM-------    457
                      .|.
CG4928-PC        486 RKYKISFFLS    495
```

Global pair-wise alignment focusing on the carboxy terminal against the whole D. melanogaster protein sequences

- Human UNC93A and CG4928-PA

Identity: 6/541 (1.1 %)
Similarity: 14/541 (2.6 %)
Gaps: 516/541 (95.4 %)

```
Human              1 -------CVESKNPIRPHAPGQVNQAEDEEIQTKM---------------     28
                            ..|:..|::|.|..:.:.|   .::.|:
CG4928-PA          1 MTGFTNAGFENDEPVKPKAGFEPDTA---SLREKVVLNPGEKWRILKNIS     47
```

- Human UNC93A and CG4928-PB

Identity: 6/541 (1.1 %)
Similarity: 14/541 (2.6 %)
Gaps: 516/541 (95.4 %)

```
Human              1 -------CVESKNPIRPHAPGQVNQAEDEEIQTKM---------------     28
                            ..|:..|::|.|..:.:.|   .::.|:
CG4928-PB          1 MTGFTNAGFENDEPVKPKAGFEPDTA---SLREKVVLNPGEKWRILKNIS     47
```

- Human UNC93A and CG4928-PC

Identity: 6/498 (1.2 %)
Similarity: 14/498 (2.8 %)
Gaps: 473/498 (95.0 %)

```
Human              1 -------CVESKNPIRPHAPGQVNQAEDEEIQTKM---------------     28
                            ..|:..|::|.|..:.:.|   .::.|:
CG4928-PC          1 MTGFTNAGFENDEPVKPKAGFEPDTA---SLREKVVLNPGEKWRILKNIS     47
```

Local pair-wise alignment focusing on the carboxy terminal against the whole D. melanogaster protein sequences

- Human UNC93A and CG4928-PA

Identity: 4/10 (40.0 %)
Similarity: 7/10 (70.0 %)
Gaps: 0/10 (0.0 %)

```
Human              3 ESKNPIRPHA     12
                     |:..|::|.|
CG4928-PA         10 ENDEPVKPKA     19
```

- Human UNC93A and CG4928-PB

Identity: 4/10 (40.0 %)
Similarity: 7/10 (70.0 %)
Gaps: 0/10 (0.0 %)

```
Human              3 ESKNPIRPHA     12
                     |:..|::|.|
CG4928-PB         10 ENDEPVKPKA     19
```

- Human UNC93A and CG4928-PC

Identity: 4/10 (40.0 %)
Similarity: 7/10 (70.0 %)
Gaps: 0/10 (0.0 %)

```
Human              3 ESKNPIRPHA     12
                     |:..|::|.|
CG4928-PC         10 ENDEPVKPKA     19
```
